# Supplementary material for: Genome-wide association analysis of plasma lipidome identifies 495 genetic associations
Source: Nat Commun. 2023 Oct 31;14:6934. doi: 10.1038/s41467-023-42532-8 (PMC10618167; doi:10.1038/s41467-023-42532-8)
Supplement: Supplementary file 1 — Supplementary Information [file 41467_2023_42532_MOESM1_ESM.pdf]

Supplementary Information

Supplementary Notes

Supplementary Note 1: Rare variants reaching high PIP only in multivariate fine-mapping and low PIP in standard lipids fine-mapping in UKB

Three rare variants with CADD score > 10, rs201563586 (MAF=0.002), rs186249276 (MAF=0.004) and rs1292311927 (MAF=0.004), reached a PIP > 0.9 in multivariate analysis but not in any univariate analysis and had PIP < 0.001 in standard lipids fine-mapping in UKBB.
The *LIPC* missense variant rs201563586 reached a PIP of 0.999838 for cluster 2 and a *P*-value of 8e-8. The lead SNP of the GWAS region, rs10468017, is not rare (MAF=0.338). In univariate fine-mapping the variant is the representative variant for uninformative credible sets of PE 16:0;0_20:4;0 (PIP=0.9119, *P* =2e-8) and PE 18:0;0_20:4;0 (PIP=0.9217, *P* =3e-8).
The *ABHD3* missense variant rs186249276 reached high PIPs of 0.9681 and 0.9333 for cluster 2 and cluster 3. The lead variant of the GWAS region, rs181026394, is also rare (MAF=0.007) but reached a low *P*-value in univariate GWAS of a trait included in cluster 2 (*P*=7e-20 for PC 14:0;0_18:2;0) and a trait included in cluster 3 (*P*=6e-11 for PC 16:1;0_18:1;0) so no simulation was performed for this variant. The missense variant rs186249276 reaches *P*-values of 4e-19 and 5e-12 for cluster 2 and cluster 3, respectively. The variant is a representative variant for PC 14:0;0_18:1;0 (PIP=0.3838, *P*=2e-10) and for an uninformative credible set of PC 14:0;0_18:2;0 (PIP=0.4485, *P*=6e-8). These lipid species are included in cluster 2, however the variant was not among the representative variants of traits included in cluster 3. The region was only fine-mapped for one trait included in cluster 3, PC 16:1;0_18:1;0 (PIP = 0.004, *P*=2e-2).
The *ELOVL2* splice region variant rs1292311927reached a PIP of 0.9898 and a *P*-value of 2e-7 for cluster 6. The lead variant of the GWAS, rs9468401, is not rare (MAF=0.153) and reached a *P*-value of 6e-20. The lowest univariate *P*-value of the lead variant for a univariate trait of cluster 6 was 1e-5 for CE 22:6;0. The region was not fine-mapped for any univariate GWAS.

Supplementary Note 2: Correlation with standard lipids of traits for which high PIP variants were found by FINEMAP

We classified the variants for which high PIP variants were found by FINEMAP by their UKB fine-mapping results into 4 categories: (1) variants with CADD score > 10 and PIP ≤ 0.1 in UKB, (2) variants with CADD score > 10 and PIP > 0.1 in UKB, (3) variants with CADD score ≤ 10 and PIP ≤ 0.1 in UKB, (4) variants with CADD score ≤ 10 and PIP > 0.1 in UKB.

To compare the correlation between standard lipids and lipid species or LCP-phenotypes, we defined the maximum correlation *mrt* between trait *t* and standard lipids as:
(1) $\mathrm{mrt} = \max(|\mathrm{corr}(t, \mathrm{HDLC})|, |\mathrm{corr}(t, \mathrm{LDLC})|, |\mathrm{corr}(t, \mathrm{TG})|, |\mathrm{corr}(t, \mathrm{TC})|)$
We calculated the maximum correlation mrv for a variant *v* as:
(2) $\mathrm{mrv} = \max(\mathrm{MRT})$,
with MRT being a list of all mrt values for traits *t* associated with *v*. We calculated the mean mrv for variants included in each category. The mean mrv was lower for variants reaching PIP ≤ 0.1 in UKB (category 1: 0.22, category 3: 0.19) compared to those reaching PIP > 0.1 in UKB (category 2: 0.66, category 4: 0.30). Wilcoxon rank sum test of the two groups had a *P*-value of 0.01632.

Supplementary Note 3: FinnGen ethics statement

Patients and control subjects in FinnGen provided informed consent for biobank research, based on the Finnish Biobank Act. Alternatively, separate research cohorts, collected prior the Finnish Biobank Act came into effect (in September 2013) and start of FinnGen (August 2017), were collected based on study-specific consents and later transferred to the Finnish biobanks after approval by Fimea (Finnish Medicines Agency), the National Supervisory Authority for Welfare and Health. Recruitment protocols followed the biobank protocols approved by Fimea. The Coordinating Ethics Committee of the Hospital District of Helsinki and Uusimaa (HUS) statement number for the FinnGen study is Nr HUS/990/2017.

The FinnGen study is approved by Finnish Institute for Health and Welfare (permit numbers: THL/2031/6.02.00/2017, THL/1101/5.05.00/2017, THL/341/6.02.00/2018, THL/2222/6.02.00/2018, THL/283/6.02.00/2019, THL/1721/5.05.00/2019 and THL/1524/5.05.00/2020), Digital and population data service agency (permit numbers: VRK43431/2017-3, VRK/6909/2018-3, VRK/4415/2019-3), the Social Insurance Institution (permit numbers: KELA 58/522/2017, KELA 131/522/2018, KELA 70/522/2019, KELA 98/522/2019, KELA 134/522/2019, KELA 138/522/2019, KELA 2/522/2020, KELA 16/522/2020), Findata permit numbers THL/2364/14.02/2020, THL/4055/14.06.00/2020,,THL/3433/14.06.00/2020, THL/4432/14.06/2020, THL/5189/14.06/2020, THL/5894/14.06.00/2020, THL/6619/14.06.00/2020, THL/209/14.06.00/2021, THL/688/14.06.00/2021, THL/1284/14.06.00/2021, THL/1965/14.06.00/2021, THL/5546/14.02.00/2020, THL/2658/14.06.00/2021, THL/4235/14.06.00/202, Statistics Finland (permit numbers: TK-53-1041-17 and TK/143/07.03.00/2020 (earlier TK-53-90-20) TK/1735/07.03.00/2021, TK/3112/07.03.00/2021) and Finnish Registry for Kidney Diseases permission/extract from the meeting minutes on 4th July 2019.

The Biobank Access Decisions for FinnGen samples and data utilized in FinnGen Data Freeze 9 include: THL Biobank BB2017_55, BB2017_111, BB2018_19, BB_2018_34, BB_2018_67, BB2018_71, BB2019_7, BB2019_8, BB2019_26, BB2020_1, Finnish Red Cross Blood Service Biobank 7.12.2017, Helsinki Biobank HUS/359/2017, HUS/248/2020, Auria Biobank AB17-5154 and amendment #1 (August 17 2020), AB20-5926 and amendment #1 (April 23 2020) and it´s modification (Sep 22 2021), Biobank Borealis of Northern Finland_2017_1013, Biobank of Eastern Finland 1186/2018 and amendment 22 § /2020, Finnish Clinical Biobank Tampere MH0004 and amendments (21.02.2020 & 06.10.2020), Central Finland Biobank 1-2017, and Terveystalo Biobank STB 2018001 and amendment 25th Aug 2020.

Supplementary Note 4: Simulation of multivariate *P*-values under the null hypothesis

We simulated multivariate *P*-values for four SNPs with minor allele frequency (MAF) 0.002, 0.012, 0.052, and 0.084 using 100,000 permutations of the genotypes. The simulations were repeated with another set of 100,000 permutations. The QQ-plots showed inflated *P*-values, which are located outside the 95% confidence interval, for some of the clusters for the rare SNP (MAF < 0.01) and the low-frequency SNP (0.01 ≤ MAF < 0.05) and no inflation for the common SNPs (MAF > 0.05). From the simulations, we conclude that multivariate *P*-values of rare or low-frequency variants can be inflated and therefore we perform these simulations for all rare or low-frequency variants reaching genome-wide significance (GWS) in a multivariate analysis, which did not reach GWS in a univariate GWAS. This was the case for one rare variant and four low-frequency variants.

We then corrected the multivariate *P*-values by the mean of the genomic inflation factors (λ), estimated from the simulations as the ratio of the median of the observed chi-square association statistics to its expectation under the null, if the simulations showed inflated *P*-values. More specifically, we divided the X^2-values by the mean λ over the two simulations and recalculated the *P*-values from the corrected X^2-values. We considered the simulated *P*-values to be inflated if the proportion of *P*-values < 0.01 was greater than 1.2%.

Empirical cumulative distribution function (ECDF)-plots for the five variants are shown in Supplementary Figure 17. We summarized the genomic inflation factors, the minimum *P*-value reached in the simulations and the proportion of *P*-values < 0.01 before and after correcting the *P*-values in Supplementary Table 4. For the first three variants rs77024033, rs4986970 and rs61751862, the proportion of *P*-values < 0.01 was greater than 1.2% and the *P*-values were therefore corrected. The rare variant rs77024033 is not GWS after the correction and is not reported as a GWS variant.

Supplementary Note 5: Supplemental acknowledgments

We want to acknowledge the participants and investigators of the FinnGen study. The FinnGen project is funded by two grants from Business Finland (HUS 4685/31/2016 and UH 4386/31/2016) and the following industry partners: AbbVie Inc., AstraZeneca UK Ltd, Biogen MA Inc., Bristol Myers Squibb (and Celgene Corporation & Celgene International II Sàrl), Genentech Inc., Merck Sharp & Dohme LCC, Pfizer Inc., GlaxoSmithKline Intellectual Property Development Ltd., Sanofi US Services Inc., Maze Therapeutics Inc., Janssen Biotech Inc, Novartis AG, and Boehringer Ingelheim International GmbH. Following biobanks are acknowledged for delivering biobank samples to FinnGen: Auria Biobank [www.auria.fi/biopankki], THL Biobank [www.thl.fi/biobank], Helsinki Biobank [www.helsinginbiopankki.fi], Biobank Borealis of Northern Finland [https://www.ppshp.fi/Tutkimus-ja-opetus/Biopankki/Pages/Biobank-Borealis-briefly-in-English.aspx], Finnish Clinical Biobank Tampere [www.tays.fi/en-US/Research_and_development/Finnish_Clinical_Biobank_Tampere], Biobank of Eastern Finland [www.ita-suomenbiopankki.fi/en], Central Finland Biobank [www.ksshp.fi/fi-FI/Potilaalle/Biopankki], Finnish Red Cross Blood Service Biobank [http://www.veripalvelu.fi/verenluovutus/biopankkitoiminta], Terveystalo Biobank [www.terveystalo.com/fi/Yritystietoa/Terveystalo-Biopankki/Biopankki/] and Arctic Biobank [https://www.oulu.fi/en/university/faculties-and-units/faculty-medicine/northern-finland-birth-cohorts-and-arctic-biobank]. All Finnish Biobanks are members of BBMRI.fi infrastructure [www.bbmri.fi]. Finnish Biobank Cooperative -FINBB [https://finbb.fi/] is the coordinator of BBMRI-ERIC operations in Finland. The Finnish biobank data can be accessed through the Fingenious® services [https://site.fingenious.fi/en/] managed by FINBB.

Supplementary Tables

Supplementary Table 1. Loci reaching genome-wide significance.

Locus	Chr	Mv trait	Mv lead variant	Mv minP	Uv trait	Uv lead variant	Uv minP	HDL	LDL	TC	TG	LS
PBX1	1	8	rs144944992	3e-4	CE 18:0;0	rs144944992	4e-8					
LINC01320	2	6	rs955533958	2e-7	CE 20:5;0	rs955533958	1e-8					
RMDN2	2	2	rs182370326	1e-2	PI 16:0;0_18:2;0	rs182370326	4e-8					
MAP4K4	2	3	rs61008604	2e-6	PC 16:0;0_22:4;0	rs61008604	5e-9					
AC092646.2	2	11	rs6709442	7e-6	PC O-16:0;0/20:4;0	rs6709442	3e-8					
PARD3B	2	4	rs149768482	5e-3	LPC 18:2;0	rs149768482	1e-8					
AC011233.1	2	5	rs138313489	5e-8	PI 18:1;0_18:1;0	rs138313489	2e-4					
LINC02005	3	2	rs1204981098	1e-4	PE 16:0;0_18:2;0	rs1204981098	5e-8					
SORCS2	4	10	rs138588594	4e-4	PC O-18:2;0/16:0;0	rs138588594	3e-8					
PCDH10	4	3	rs62311841	4e-6	PC O-16:0;0/16:1;0	rs62311841	3e-8					
MIR548A1HG	6	5	rs72021940	2e-7	PC 18:1;0_18:1;0	rs72021940	2e-9					
MIR3144	6	7	rs184149665	2e-7	CE 16:0;0	rs184149665	2e-8					
CNTNAP2	7	2	rs149545162	1e-3	PE 18:1;0_18:1;0	rs149545162	1e-8					
DPP6	7	3	rs1391507096	3e-5	PC 18:2;0_20:4;0	rs1391507096	1e-8					
TOX	8	1	rs7846649	5e-4	TAG 53:4;0	rs7846649	2e-8					
SAMD12	8	8	rs28505920	9e-4	Cer 40:1;2	rs28505920	4e-8					
EMG1	12	11	rs2041601	2e-8	PC O-16:1;0/20:4;0	rs2041601	1e-6					X
SOX5	12	10	rs11047412	4e-8	PC O-16:0;0/16:0;0	rs11047412	6e-5					
AC090017.1	12	2	rs73120814	4e-5	PE 18:0;0_18:2;0	rs73120814	4e-8					
LINC01619	12	2	rs73215839	1e-4	PC 18:0;0_18:3;0	rs73215839	4e-8					
SCARB1	12	11	rs11057853	1e-4	PC O-18:1;0/20:4;0	rs11057853	2e-8	X				
CAB39L	13	4	rs75654243	1e-4	LPC 18:2;0	rs75654243	2e-8					
ABHD2	15	3	rs293396	8e-9	PC 18:2;0_20:4;0	rs1033155	2e-8	X			X	
MSRB1	16	7	rs147549994	7e-6	PC 18:0;0_20:4;0	rs147549994	1e-8					
ZFHX3	16	2	rs117210444	1e-5	PI 18:1;0_20:4;0	rs117210444	9e-9					
G6PC	17	8	rs201961848	6e-5	Cer 40:1;2	rs201961848	4e-8					
AQP4-AS1	18	7	rs62081852	9e-5	PC 16:0;0_20:2;0	rs62081852	9e-9					
DCC	18	3	rs147797702	5e-7	PC 18:1;0_20:4;0	rs147797702	4e-8					
UCA1	19	7	rs55660824	3e-5	CE 18:0;0	rs55660824	5e-8		X	X		

uv univariate, *mv* multivariate, *Chr* chromosome, *minP* minimum of *P*-values of mv or uv GWAS
uv trait lipid species, *mv trait* cluster number, *LS* lipid species, *lead variant* rsid
Associations not reaching the Bonferroni corrected significance level are shown. Loci found by previous studies for standard lipids or lipid species are marked with an X. Loci are listed as italic gene names. Novel loci are bolded.

Supplementary Table 2. Representative variants from FINEMAP for novel loci.

Locus	Trait	CS	Variant	PIP	Function	Gene
DTL	cluster 5	1	rs571587214-A	0.30	intergenic	
		2	rs72747044-C*	0.16	3'-UTR	*LPGAT1*
STK39	cluster 8	1	rs143928330-G	1	intergenic	
		2	rs182274987-G	0.56	3'-UTR	*CERS6*
		3	rs6728199-T*	0.09	intergenic	
CDS1	cluster 2	1	rs191460656-T	0.62	intron	*CDS1*
AGPAT2	PC16:0;0_22:5;0	1	rs2236514-C	0.96	intron	*AGPAT2*
AGPAT2	cluster 3	1	rs2236514-C	0.99	intron	*AGPAT2*
SGPL1	Cer42:2;2	1	rs12763964-C	0.59	intron	*SGPL1*
		2	rs7091934-T*	0.05	non coding transcript exon variant	*MTND2P15*
SGPL1	cluster 8	1	rs12763964-C	1	intron	*SGPL1*
		2	rs35541340-A	0.94	upstream gene	*PCBD1*
		3	rs1538680-A*	0.27	intron	*TBATA*
YPEL2	cluster 11	1	rs149807191-T	0.30	intron	*YPEL2*
		2	rs12944342-T*	0.42	intron	*GDPD1*
		3	rs8081886-T*	0.06	intergenic	
KCNJ12	cluster 7	1	rs1320254-C	0.44	intron	*KCNJ12*
SPHK2	cluster 8	1	rs61751862-C	1	missense	*SPHK2*
		2	rs430989-G*	0.18	non coding transcript exon variant	*RPL18*
NINL	cluster 3	1	rs796307480-G	0.01	inframe deletion	*NINL*
AGPAT3	cluster 3	1	rs62229686-T	1	missense	*AGPAT3*

Variant rsid-minor allele, *Function* variant function from VEP, *Gene* gene name from VEP in italic
* variant of uninformative credible set (minimum LD < 0.1)
Representative variants (top variant or functional variant in LD with $r^2 > 0.95$) of the credible sets for the most probable number of causal variants are listed. Loci are listed as italic gene names.

Supplementary Table 3. Basic characteristics of the GeneRISK study cohort.

	Women	Men
N	4642	2624
Age (years)	55.7 (5.7)	55.9 (5.9)
BMI (kg/m2)	27.2 (5.3)	27.6 (4.4)
BMI <25 kg/m2 (*N* (%))	1771 (38.2)	731 (27.9)
BMI 25-30 kg/m2 (*N* (%))	1730 (37.3)	1253 (47.8)
BMI ≥30 kg/m2 (*N* (%))	1133 (24.5)	636 (24.3)
Diabetes (N (%))	268 (5.8)	242 (9.2)
Current smokers (*N* (%))	701 (15.1)	555 (21.2)
Lipid lowering medication (*N* (%))	432 (9.3)	373 (14.2)
LDL-C (mmol/l)	3.31 (0.94)	3.44 (0.92)
HDL-C (mmol/l)	1.79 (0.50)	1.43 (0.45)
TG (mmol/l)	1.15 (0.65)	1.49 (1.18)
TC (mmol/l)	5.61 (1.04)	5.52 (1.02)

N number
Data are listed as mean (standard deviation) or number (percentage).

Supplementary Table 4. Simulation results for rare or low-frequency variants.

variant	cluster	MAF	lambda run1 run2	*P*	Corr. *P*	minP run1 run2	Corr. minP run1 run2	Prop. *P* < 0.01 run1 run2	Corr. prop. *P* < 0.01 run1 run2
rs77024033	4	0.5%	1.06<br>1.05	3e-8	8e-8	2e-6<br>1e-6	3e-6<br>3e-6	1.27%,<br>1.32%	1.03%<br>1.10%
rs4986970	7	2.8%	1.06<br>1.06	1e-16	8e-16	4e-6<br>4e-6	7e-6<br>8e-6	1.21%<br>1.22%	0.96%<br>1.00%
rs61751862	8	3.1%	1.06<br>1.07	6e-16	5e-15	4e-6<br>6e-7	7e-6<br>1e-6	1.21%<br>1.21%	0.97%<br>0.96%
rs143928330	8	3.1%	1.05<br>1.05	3e-10	8e-10	9e-7<br>4e-6	2e-6<br>7e-6	1.14%<br>1.16%	0.97%<br>0.95%
rs116329252	5	4.2%	1.04<br>1.05	4e-11	1e-10	1e-6<br>2e-5	2e-6<br>3e-5	1.09%<br>1.15%	0.93%<br>0.97%

λ Genomic inflation factor estimated from simulation *P* multivariate *P*-value (two-sided *P*-value calculated using canonical correlation analysis) *Corr. P* multivariate *P*-value corrected by the mean of λ estimated from the simulations *minP* minimum *P*-value reached in simulation *Corr. minP* minimum *P*-value reached in simulation after correction by λ *Prop. P* < 0.01 Proportion of *P*-values which is < 0.01, *Corr. prop. P* < 0.01 Proportion of *P*-values which is < 0.01 after correction by λ

Supplementary Figures

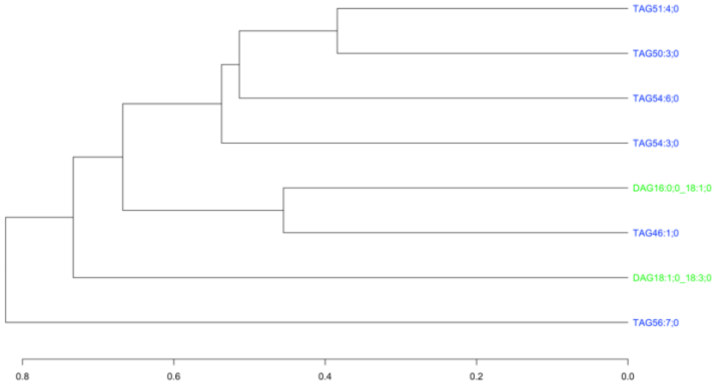

Supplementary Figure 1. Dendrogram for hierarchical clustering of lipid species of the lipid classes TAG and DAG. Only lipid species with pairwise correlations < 0.8 were included. All lipid species are contained in cluster 1.

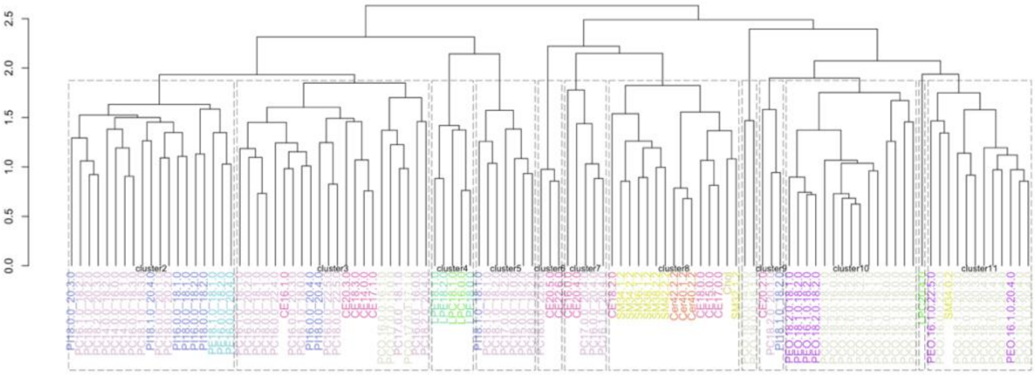

Supplementary Figure 2. Dendrogram for hierarchical clustering of lipid species of the lipid classes CE, Cer, Chol, LPC, LPE, PC, PCO, PE, PEO, PI and SM. Only lipid species with pairwise correlations < 0.8 were included. Lipid species contained in a cluster for multivariate analysis are surrounded by rectangles and labeled by cluster name.

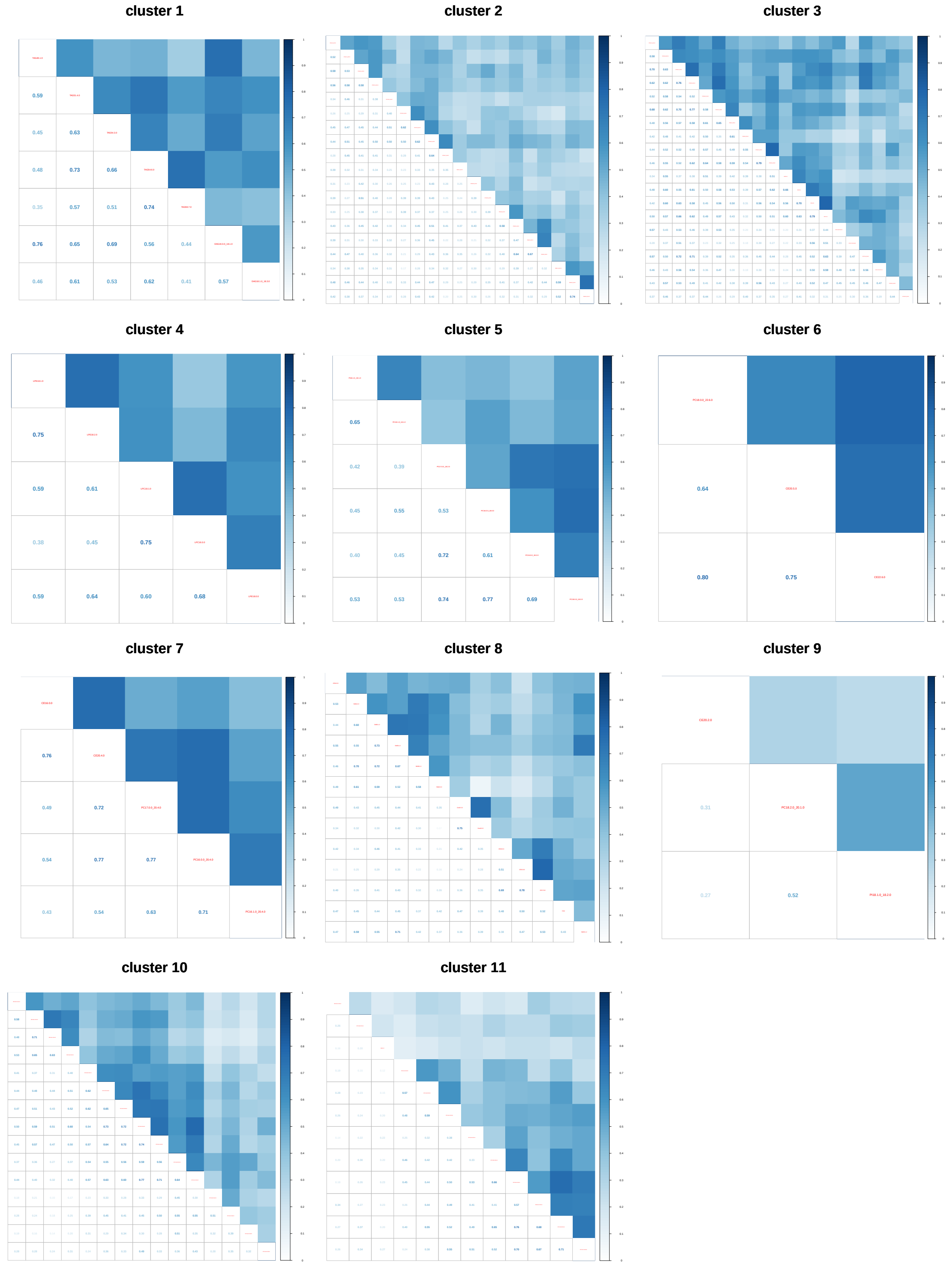

Supplementary Figure 3. Heatmaps of absolute pairwise Pearson correlations for lipid species included in a specific cluster.

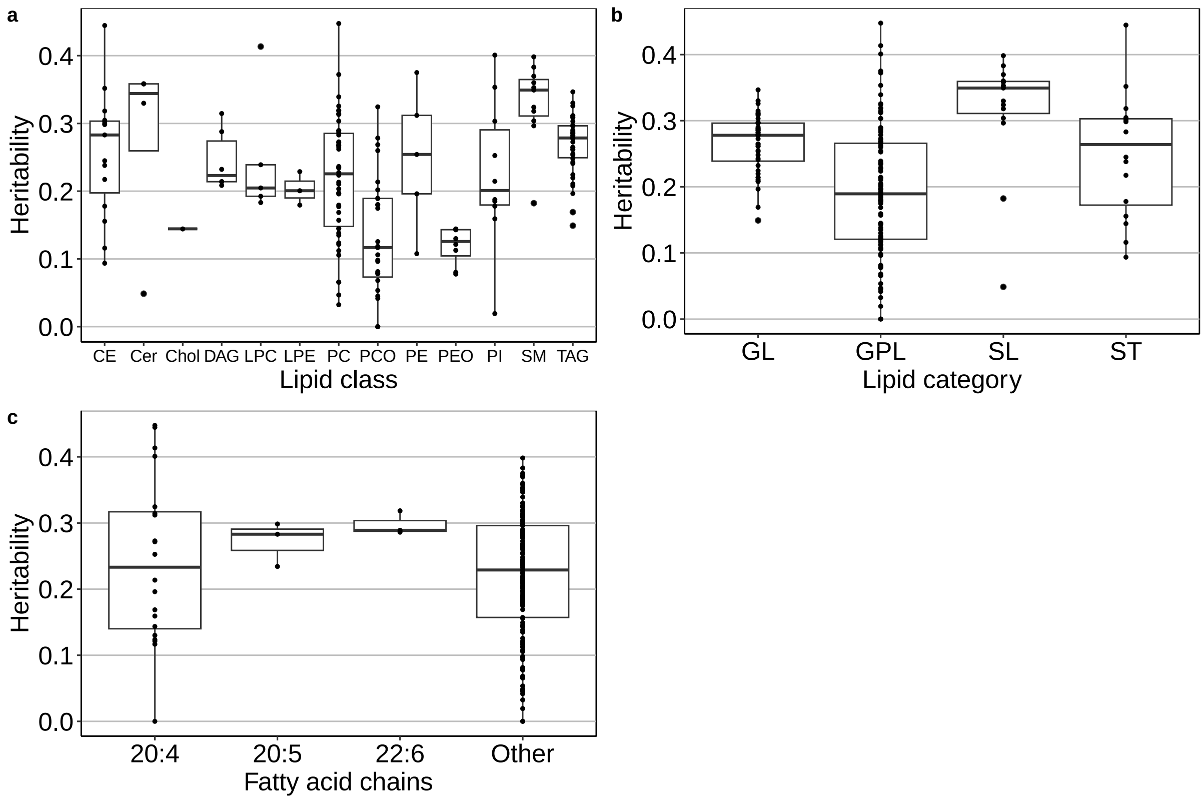

Supplementary Figure 4. Box plots of heritability estimates between species of different lipid classes (a), lipid categories (b) and species containing specific fatty acid chains (c). The boxplots depict the median (middle line), interquartile range (IQR) defining the bounds of the box, and whiskers extending to the largest/smallest values no further than 1.5 times the IQR. Data beyond the end of whiskers are plotted individually. Heritability estimation was performed using n=7,174 biologically independent samples. The number of lipid species per lipid class/lipid category/species containing specific fatty acid chains is: 15 (CE), 4 (Cer), 1 (Chol), 6 (DAG), 5 (LPC), 3 (LPE), 46 (PC), 27 (PCO), 5 (PE), 8 (PEO), 10 (PI), 11 (SM), 38 (TAG); 44 (GL), 104 (GPL), 15 (SL), 16 (ST); 20:4 (20), 20:5 (3), 22:6 (3).

CE

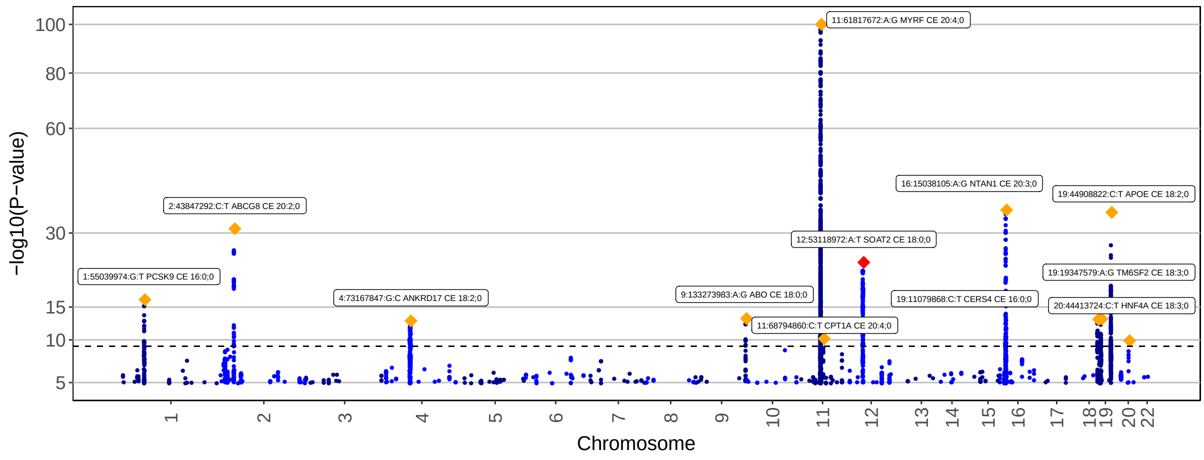

1:55039974:G:T PCSK9 CE 16:0;0
2:43847292:C:T ABCG8 CE 20:2;0
4:73167847:G:C ANKRD17 CE 18:2;0
9:133273983:A:G ABO CE 18:0;0
11:61817672:A:G MYRF CE 20:4;0
11:68794860:C:T CPT1A CE 20:4;0
12:53118972:A:T SOAT2 CE 18:0;0
16:15038105:A:G NTAN1 CE 20:3;0
19:11079868:C:T CERS4 CE 16:0;0
19:44908822:C:T APOE CE 18:2;0
19:19347579:A:G TM6SF2 CE 18:3;0
20:44413724:C:T HNF4A CE 18:3;0
−log10(P−value)
Chromosome

Cer

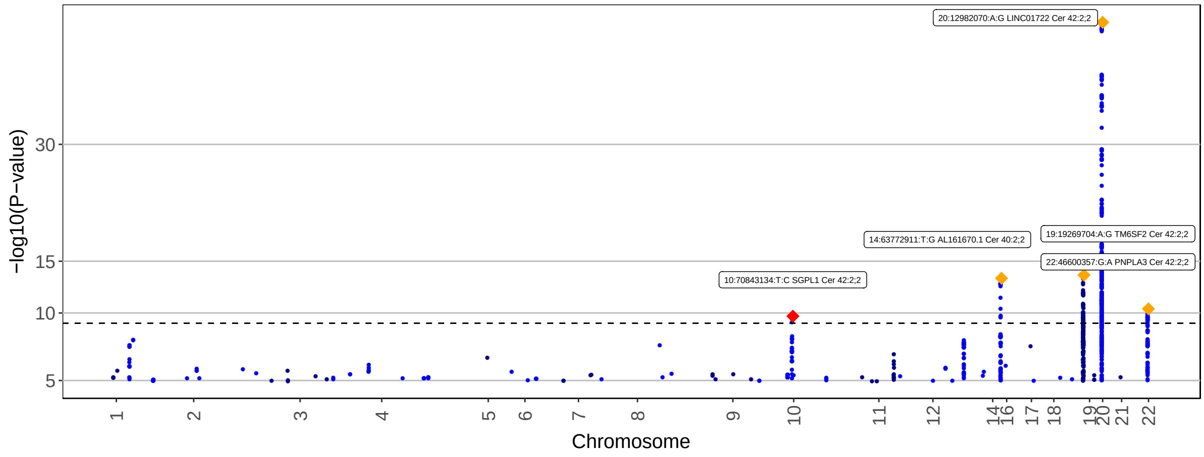

20:12982070:A:G LINC01722 Cer 42:2;2
14:63772911:T:G AL161670.1 Cer 40:2;2
19:19269704:A:G TM6SF2 Cer 42:2;2
22:46600357:G:A PNPLA3 Cer 42:2;2
10:70843134:T:C SGPL1 Cer 42:2;2
−log10(P−value)
Chromosome

Chol

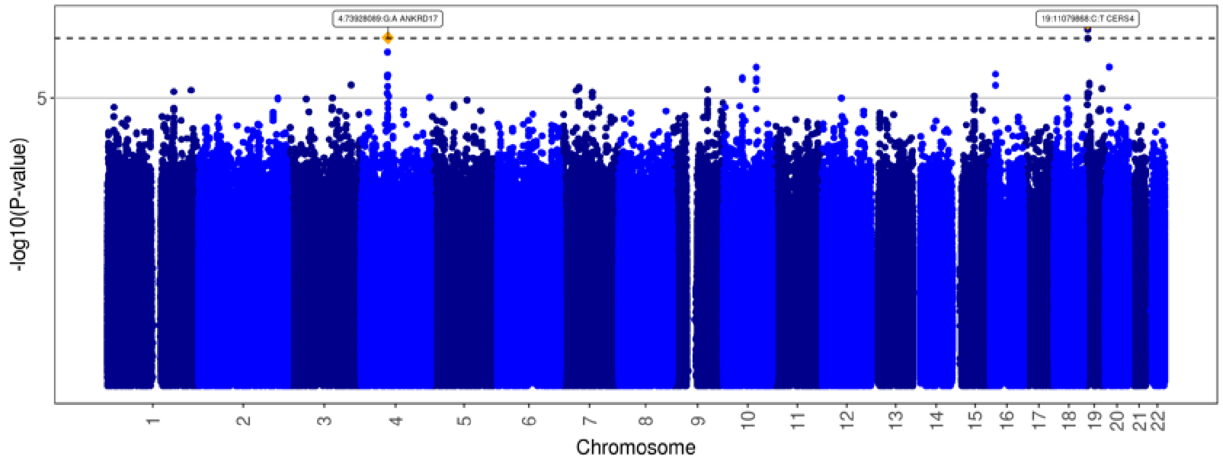

4:73928089:G:A ANKRD17
19:11079868:C:T CERS4
-log10(P-value)
Chromosome

DAG

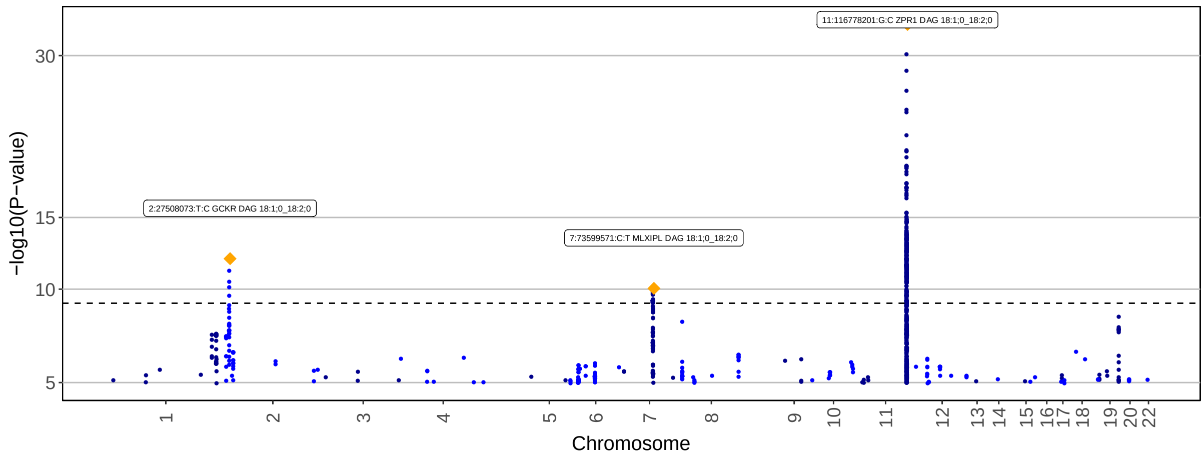
−log10(P−value)
Chromosome
11:116778201:G:C ZPR1 DAG 18:1;0_18:2;0
2:27508073:T:C GCKR DAG 18:1;0_18:2;0
7:73599571:C:T MLXIPL DAG 18:1;0_18:2;0

LPC

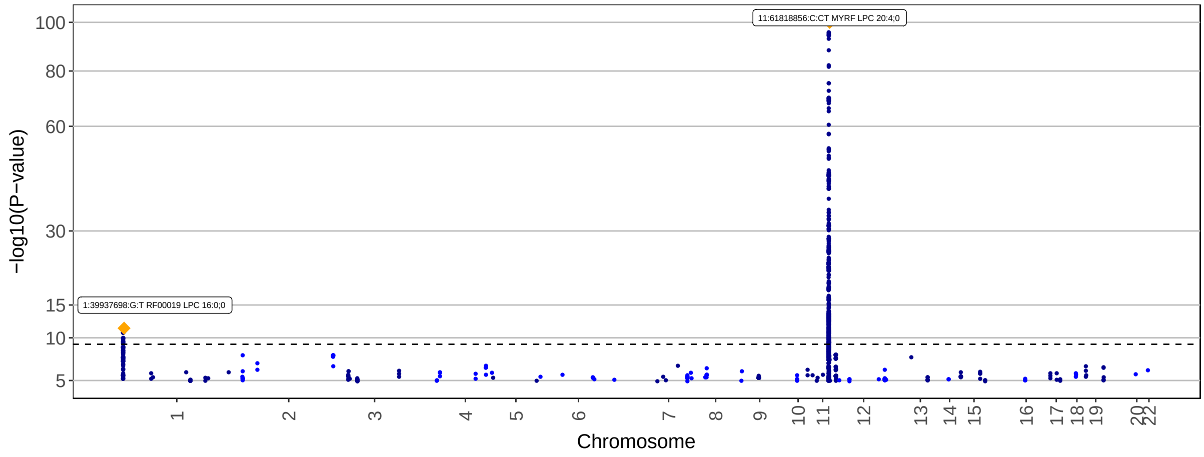
−log10(P−value)
Chromosome
11:61818856:C:CT MYRF LPC 20:4;0
1:39937698:G:T RF00019 LPC 16:0;0

LPE

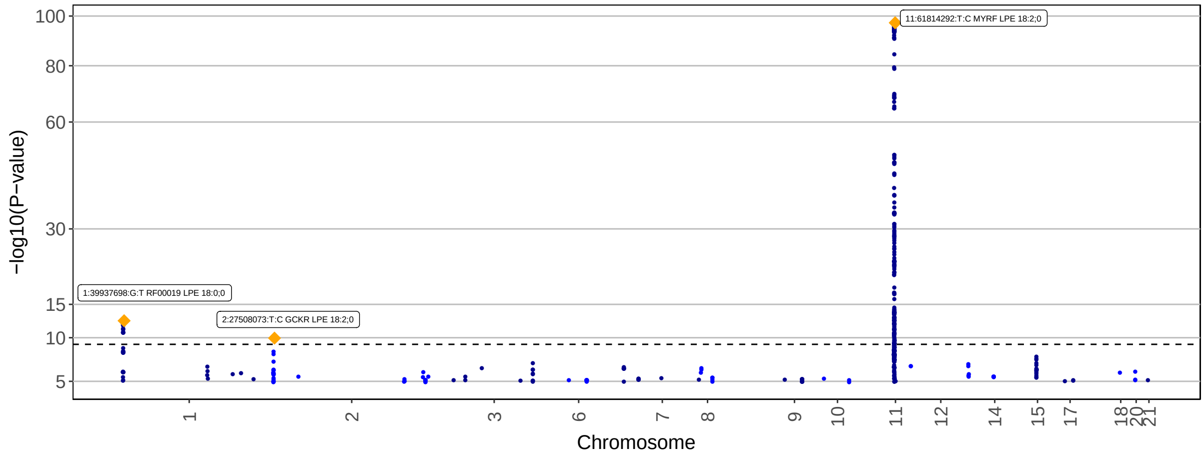
−log10(P−value)
Chromosome
11:61814292:T:C MYRF LPE 18:2;0
1:39937698:G:T RF00019 LPE 18:0;0
2:27508073:T:C GCKR LPE 18:2;0

PC

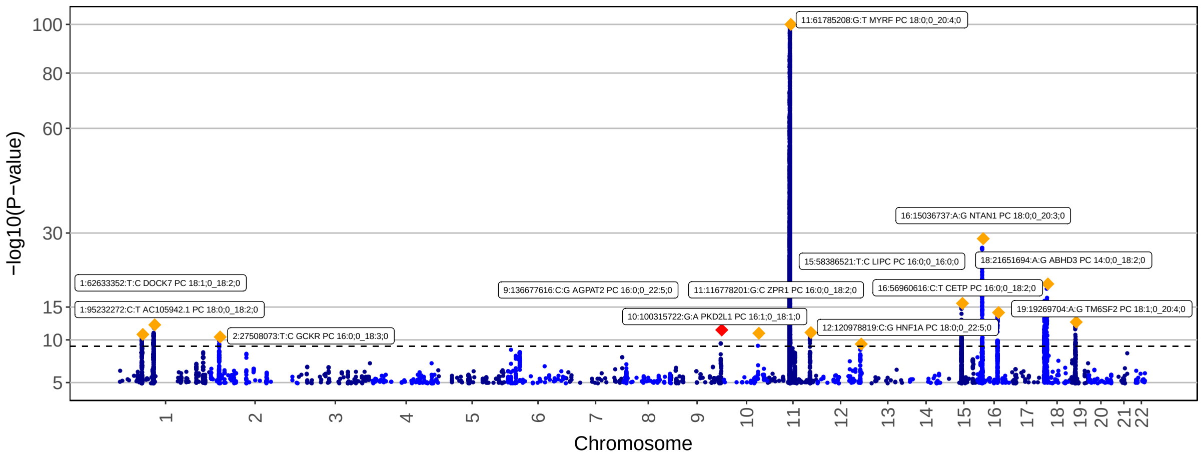
−log10(P−value)
100
80
60
30
15
10
5
11:61785208:G:T MYRF PC 18:0;0_20:4;0
16:15036737:A:G NTAN1 PC 18:0;0_20:3;0
15:58386521:T:C LIPC PC 16:0;0_16:0;0
18:21651694:A:G ABHD3 PC 14:0;0_18:2;0
1:62633352:T:C DOCK7 PC 18:1;0_18:2;0
9:136677616:C:G AGPAT2 PC 16:0;0_22:5;0
11:116778201:G:C ZPR1 PC 16:0;0_18:2;0
16:56960616:C:T CETP PC 16:0;0_18:2;0
1:95232272:C:T AC105942.1 PC 18:0;0_18:2;0
19:19269704:A:G TM6SF2 PC 18:1;0_20:4;0
10:100315722:G:A PKD2L1 PC 16:1;0_18:1;0
12:120978819:C:G HNF1A PC 18:0;0_22:5;0
2:27508073:T:C GCKR PC 16:0;0_18:3;0
1 2 3 4 5 6 7 8 9 10 11 12 13 14 15 16 17 18 19 20 21 22
Chromosome

PCO

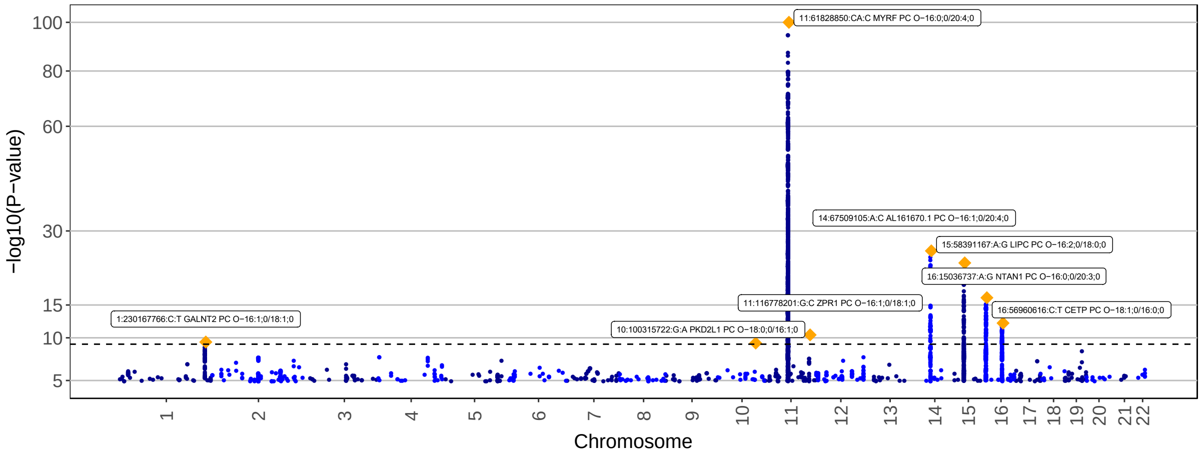
−log10(P−value)
100
80
60
30
15
10
5
11:61828850:CA:C MYRF PC O−16:0;0/20:4;0
14:67509105:A:C AL161670.1 PC O−16:1;0/20:4;0
15:58391167:A:G LIPC PC O−16:2;0/18:0;0
16:15036737:A:G NTAN1 PC O−16:0;0/20:3;0
11:116778201:G:C ZPR1 PC O−16:1;0/18:1;0
16:56960616:C:T CETP PC O−18:1;0/16:0;0
1:230167766:C:T GALNT2 PC O−16:1;0/18:1;0
10:100315722:G:A PKD2L1 PC O−18:0;0/16:1;0
1 2 3 4 5 6 7 8 9 10 11 12 13 14 15 16 17 18 19 20 21 22
Chromosome

PE

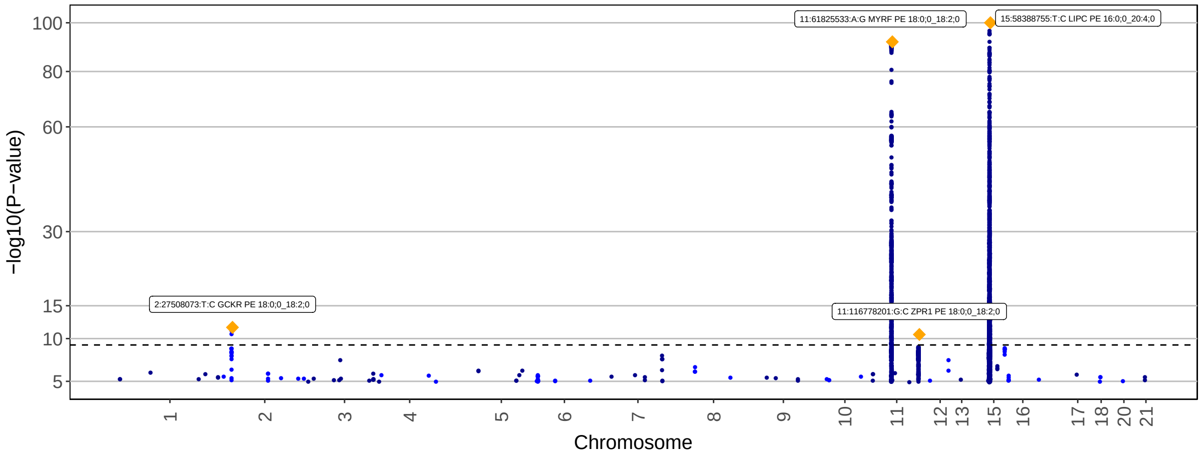
−log10(P−value)
100
80
60
30
15
10
5
11:61825533:A:G MYRF PE 18:0;0_18:2;0
15:58388755:T:C LIPC PE 16:0;0_20:4;0
2:27508073:T:C GCKR PE 18:0;0_18:2;0
11:116778201:G:C ZPR1 PE 18:0;0_18:2;0
1 2 3 4 5 6 7 8 9 10 11 12 13 15 16 17 18 20 21
Chromosome

PEO

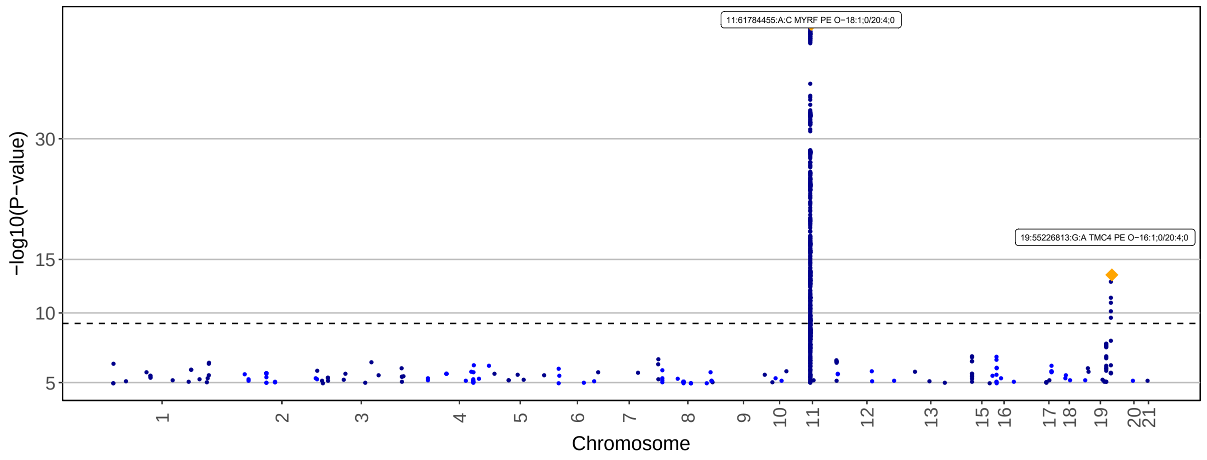

11:61784455:A:C MYRF PE O−18:1;0/20:4;0
19:55226813:G:A TMC4 PE O−16:1;0/20:4;0
−log10(P−value)
Chromosome

PI

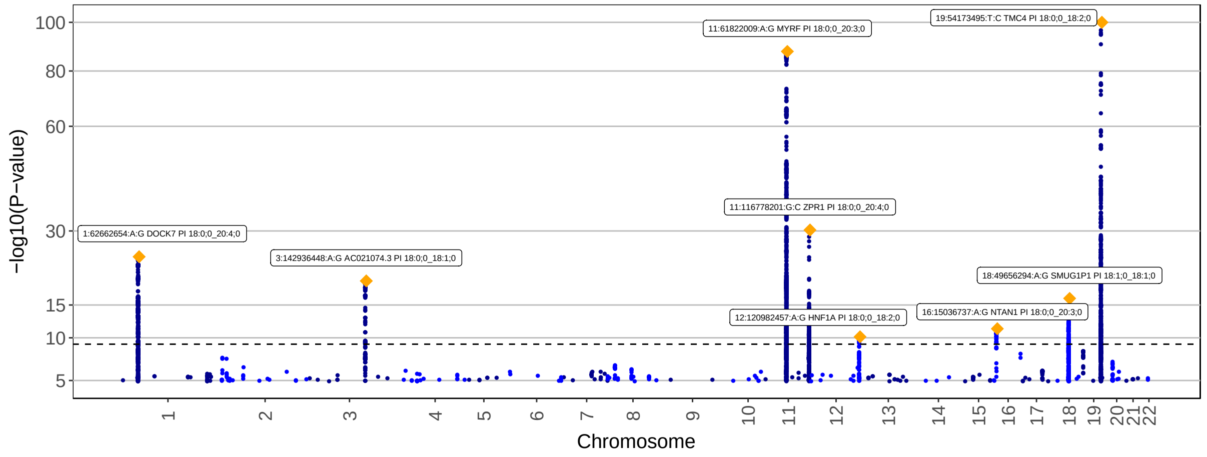

11:61822009:A:G MYRF PI 18:0;0_20:3;0
19:54173495:T:C TMC4 PI 18:0;0_18:2;0
11:116778201:G:C ZPR1 PI 18:0;0_20:4;0
1:62662654:A:G DOCK7 PI 18:0;0_20:4;0
3:142936448:A:G AC021074.3 PI 18:0;0_18:1;0
18:49656294:A:G SMUG1P1 PI 18:1;0_18:1;0
12:120982457:A:G HNF1A PI 18:0;0_18:2;0
16:15036737:A:G NTAN1 PI 18:0;0_20:3;0
−log10(P−value)
Chromosome

SM

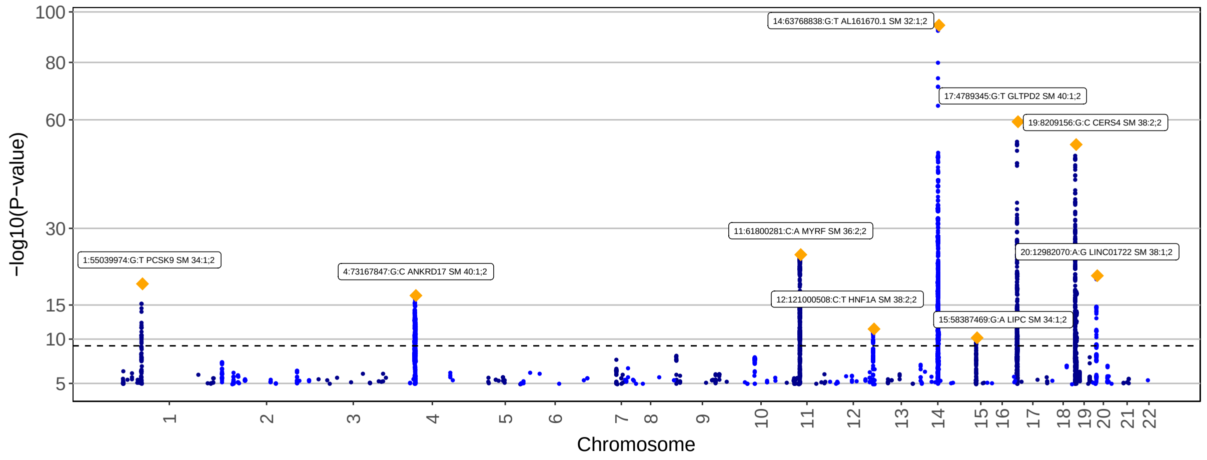

14:63768838:G:T AL161670.1 SM 32:1;2
17:4789345:G:T GLTPD2 SM 40:1;2
19:8209156:G:C CERS4 SM 38:2;2
11:61800281:C:A MYRF SM 36:2;2
20:12982070:A:G LINC01722 SM 38:1;2
1:55039974:G:T PCSK9 SM 34:1;2
4:73167847:G:C ANKRD17 SM 40:1;2
12:121000508:C:T HNF1A SM 38:2;2
15:58387469:G:A LIPC SM 34:1;2
−log10(P−value)
Chromosome

TAG

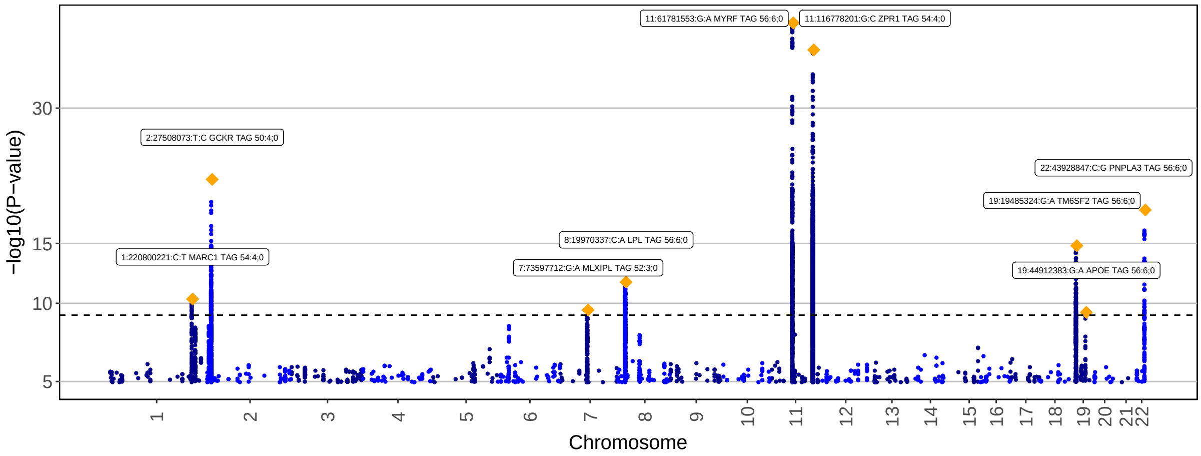

Supplementary Figure 5. Manhattan plots for lipid classes. Top associations reaching Bonferroni-corrected *P*-value threshold of 7.352941e-10 are depicted as diamonds and annotated by variant, locus name and lipid species. Known loci are colored yellow and novel loci are colored red. The vertical line indicates the Bonferroni-corrected *P*-value threshold. Only variants with *P*-value < 1e-5 are shown. The *y*-axis is capped at 100. Two-sided *P*-values calculated using a linear-mixed-model are reported.

cluster 1

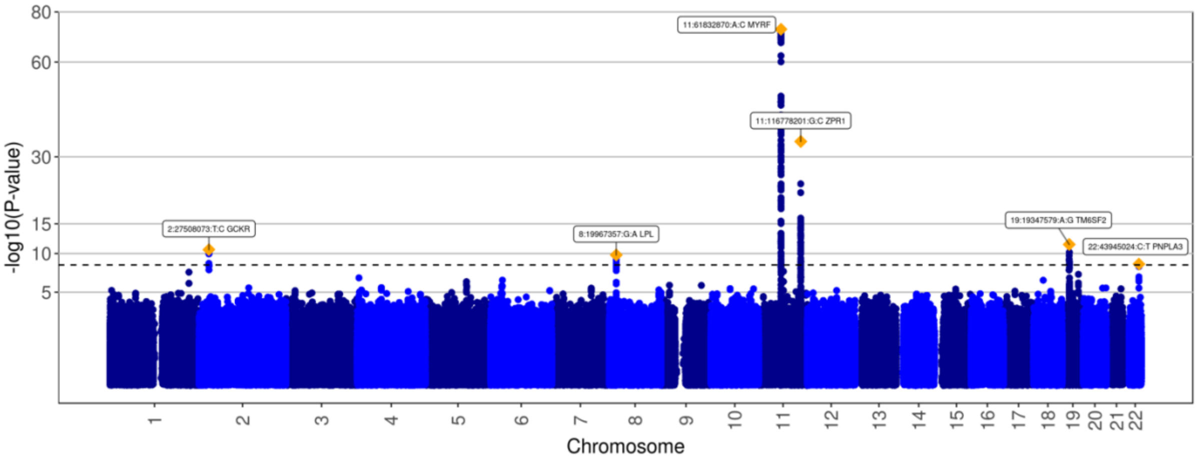

cluster 2

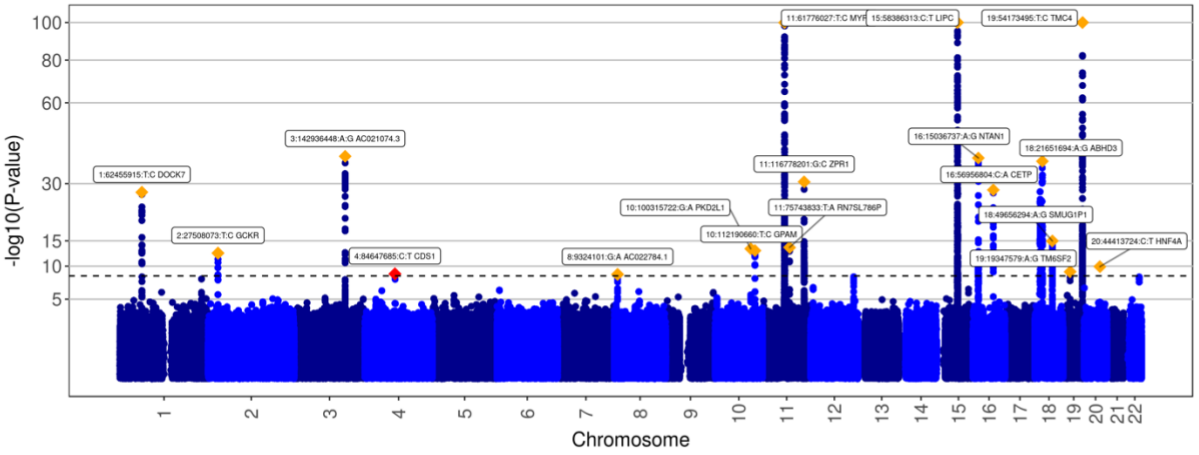

cluster 3

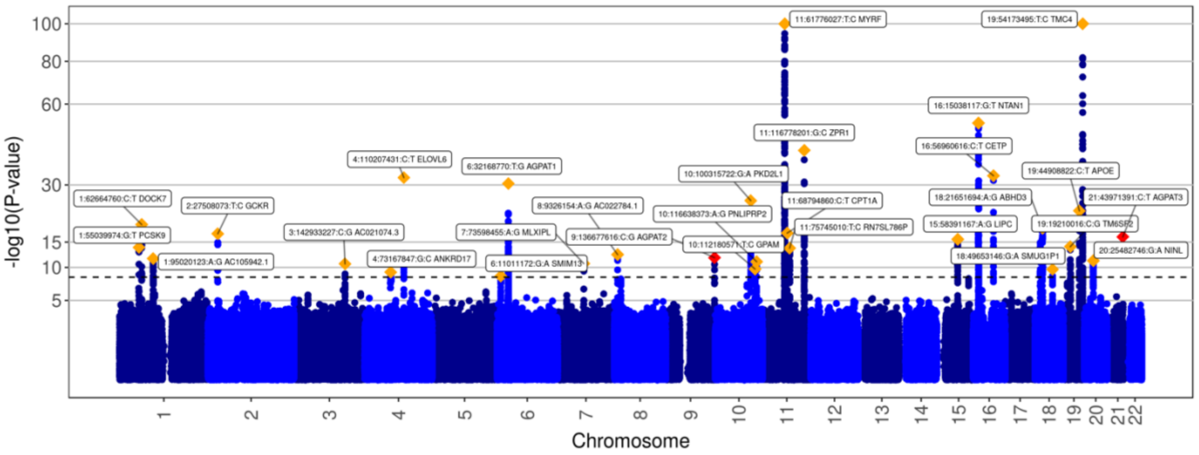

cluster 4

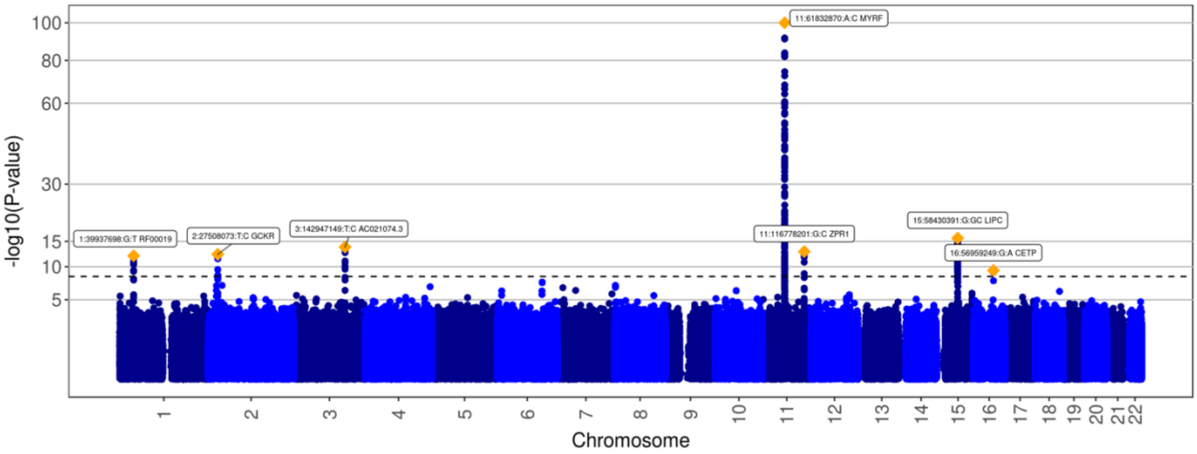

cluster 5

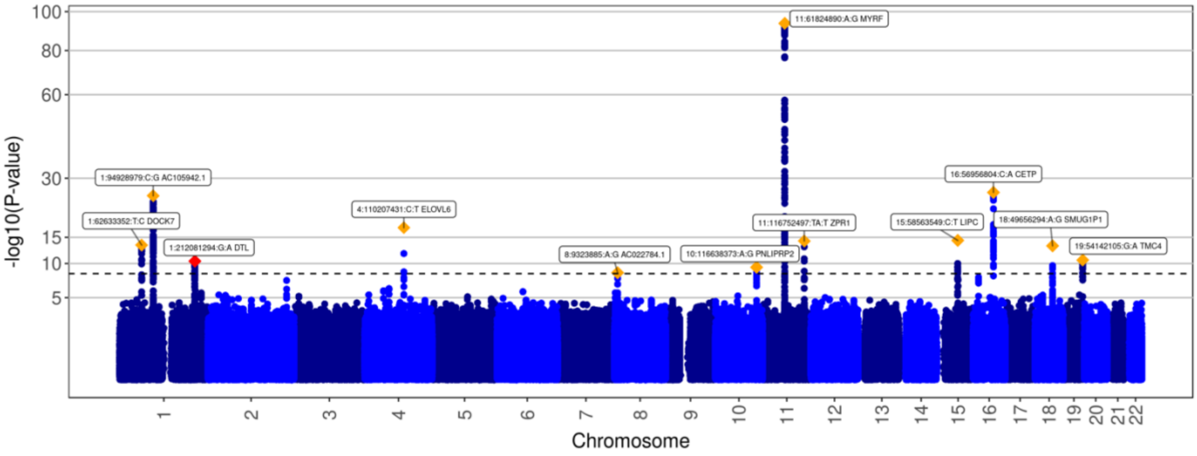

cluster 6

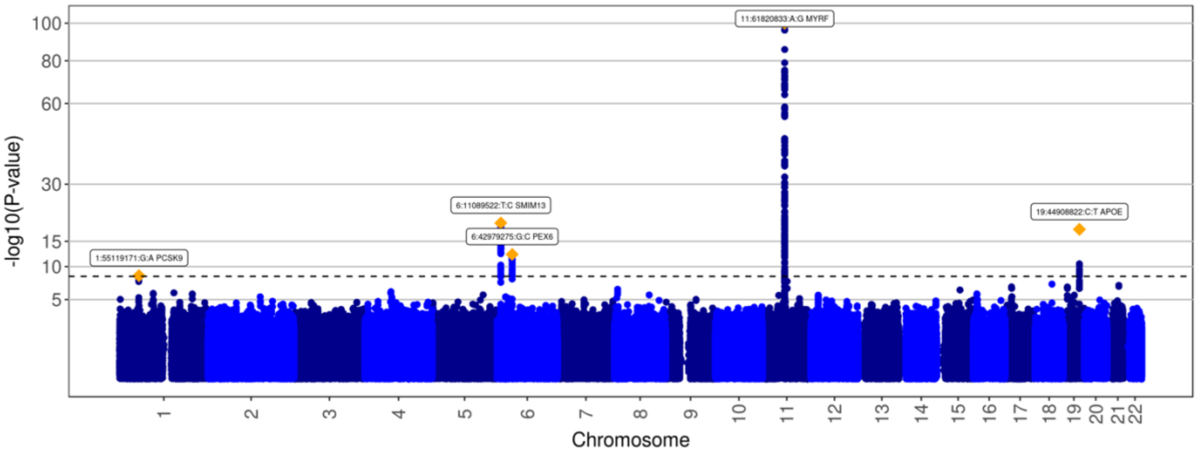

cluster 7

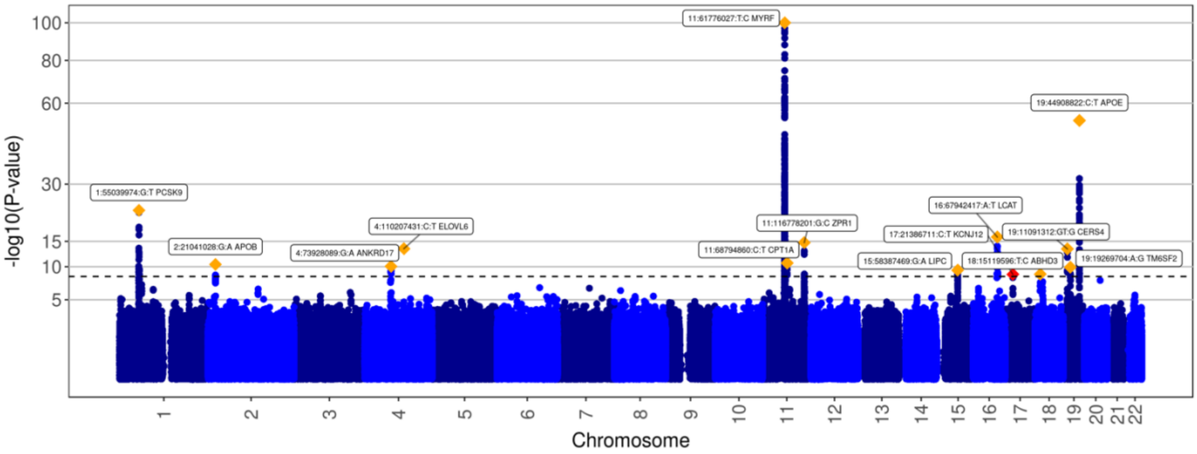

cluster 8

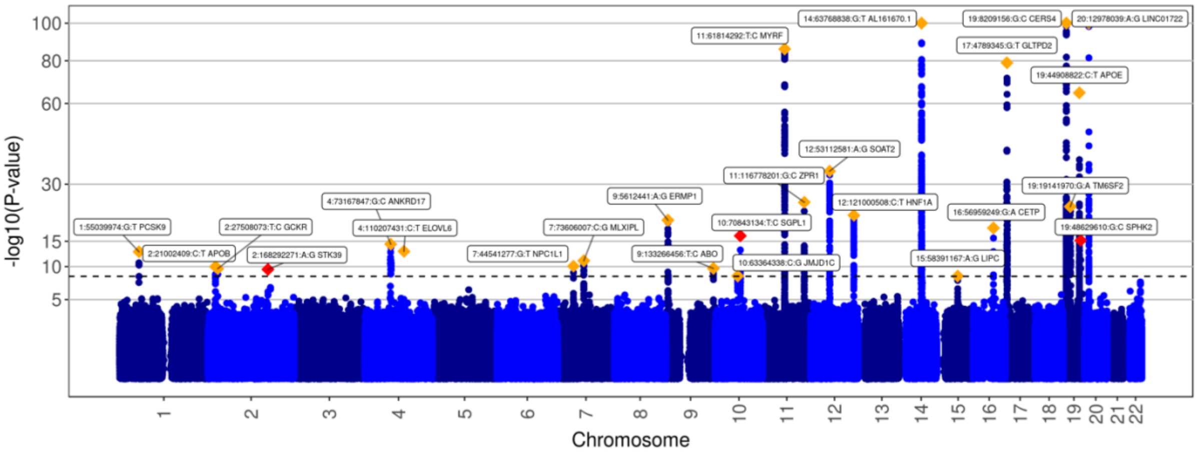

cluster 9

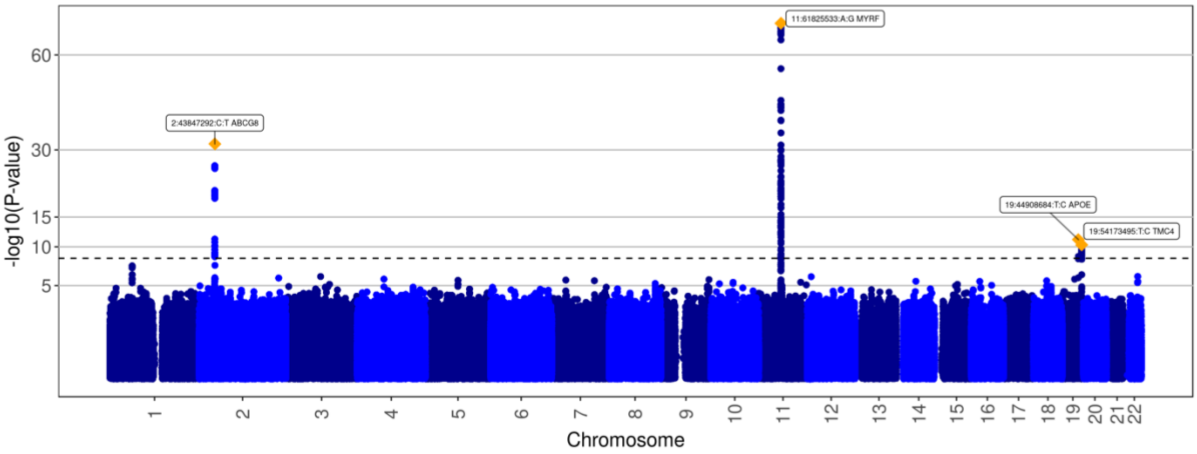

cluster 10

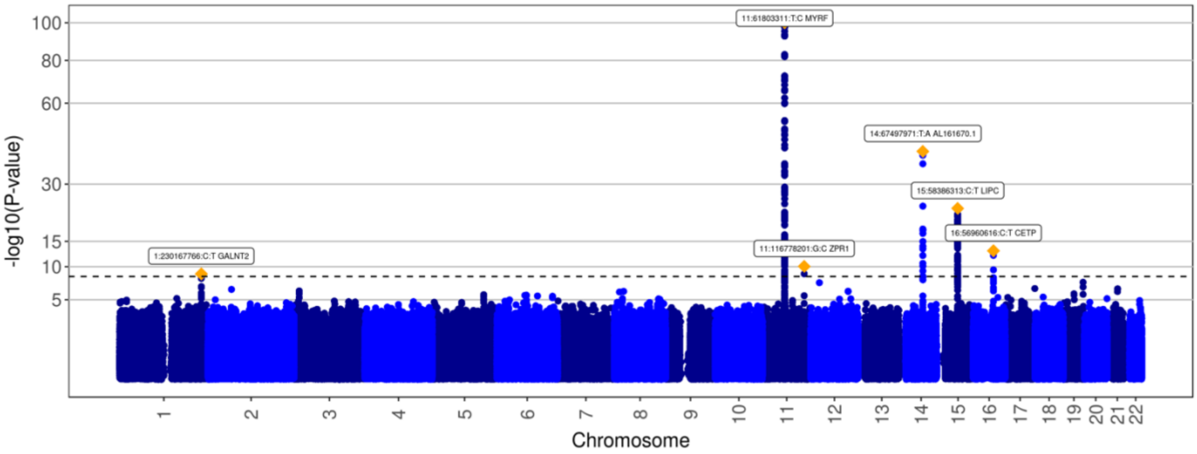

cluster 11

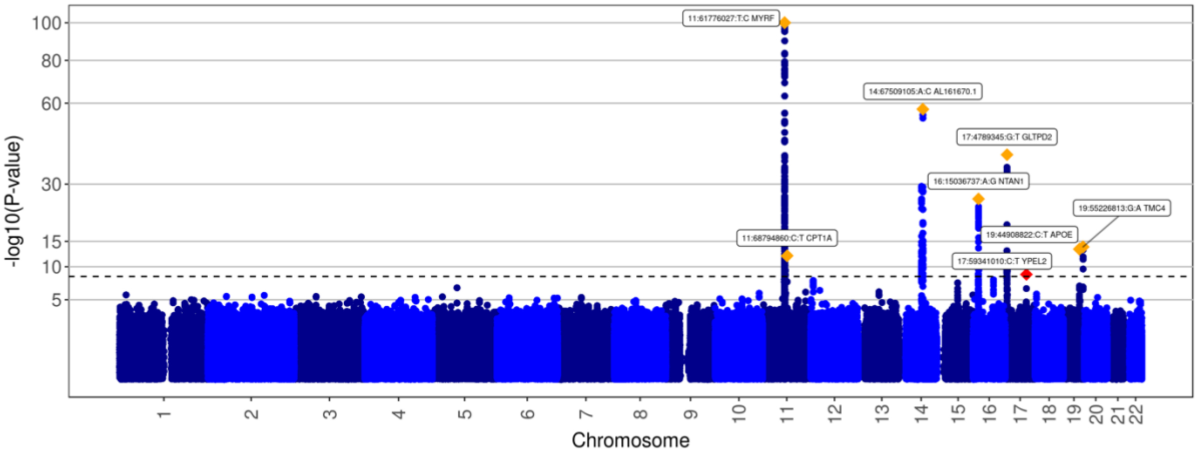

Supplementary Figure 6. Manhattan plots for multivariate clusters
Top associations for signals reaching the Bonferroni-corrected *P*-value threshold of 4.545455e-9 are depicted as diamonds and annotated by variant and locus name. Known loci are colored yellow and novel loci are colored red. The vertical line indicates the Bonferroni-corrected *P*-value threshold**.** Two-sided *P*-values calculated using canonical correlation analysis (mv) are reported.

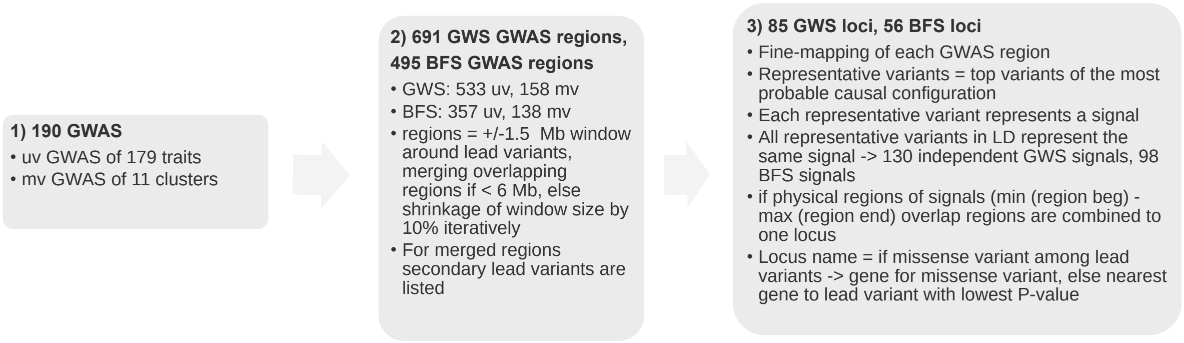

Supplementary Figure 7. Flowchart of the locus definition process. Numbers of GWAS regions and loci reaching the genome-wide significance (GWS) threshold and the Bonferroni-corrected significance (BFS) threshold are given for univariate (uv) and multivariate (mv) GWAS.

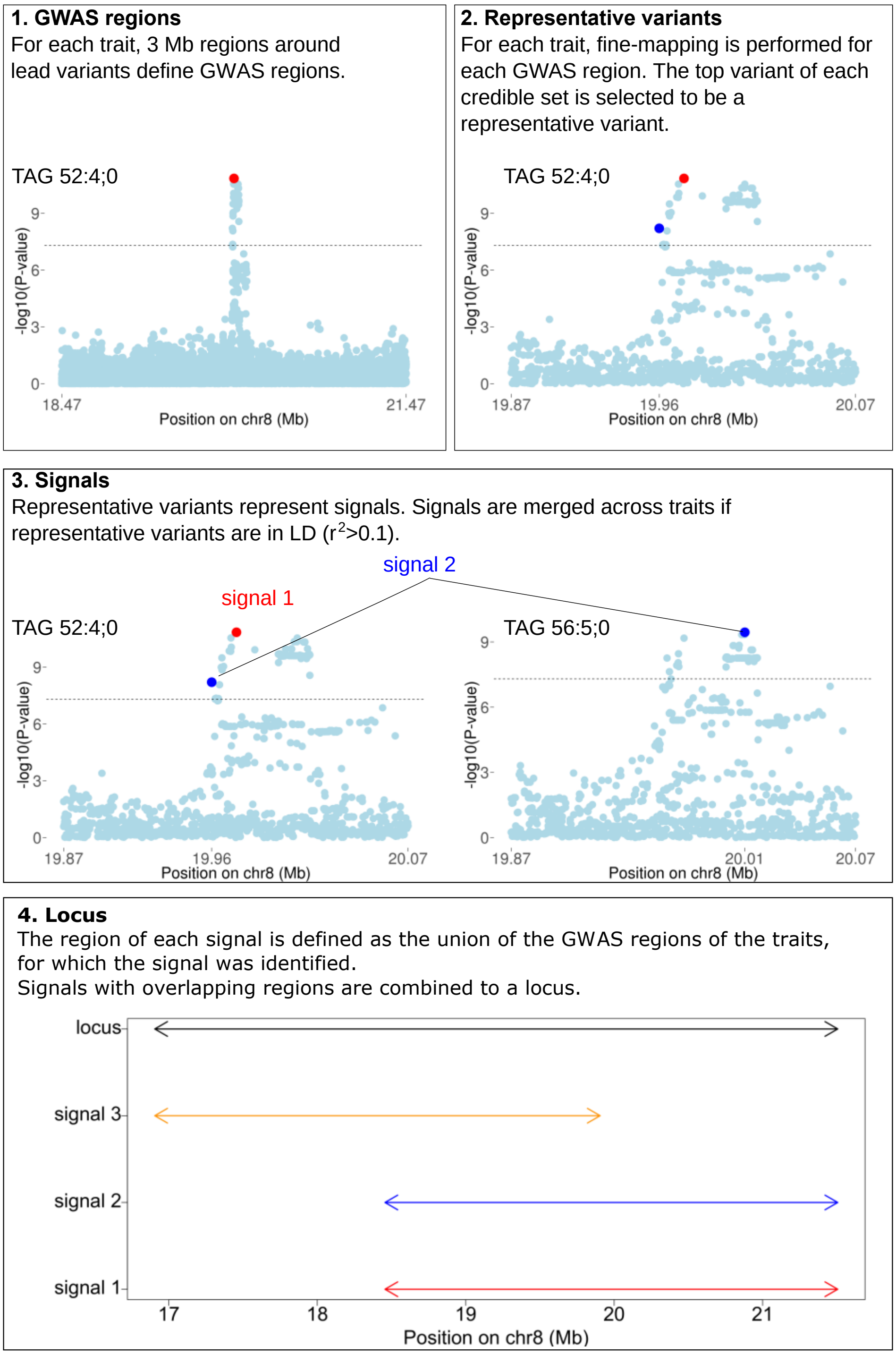

Supplementary Figure 8. Locus definition process for locus *LPL*. 1. *P*-values of variants in the 3 Mb GWAS region around lead variant (colored red) for TAG 52:4;0. Two-sided *P*-values calculated using a linear-mixed-model are shown. Not shown here: overlapping GWAS regions are merged (see Methods). 2. *P*-values of variants in 0.2 Mb window around the lead variant for TAG 52:4;0. Representative variants are colored (red and blue), the red representative variant is also the lead variant. 3. *P*-values of variants in 0.2 Mb window around the lead variant for TAG 52:4;0 and *P*-values in the same region for TAG 56:5;0. The representative variant for TAG 56:5;0 (blue) is in LD with the blue representative variant for TAG 52:4;0 and therefore represents the same signal (signal 2). The position of the representative variant of signal 2 (blue) is marked for both traits. 4. Regions of the signals and the locus.

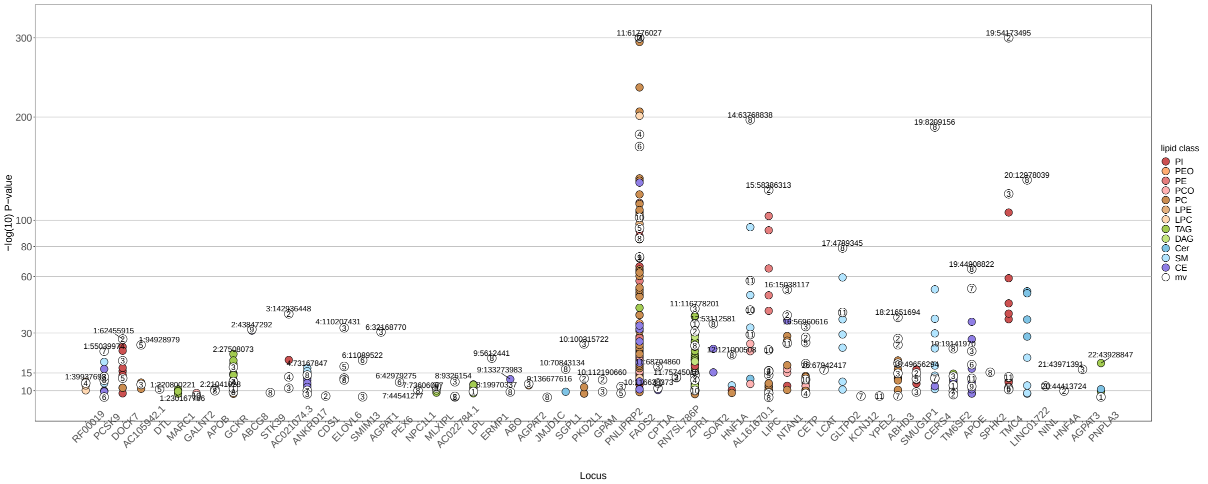

Supplementary Figure 9. Univariate and multivariate *P*-values for loci reaching Bonferroni-corrected threshold of 7.352941e-10. Loci are colored by lipid class in univariate analysis and labeled by cluster number from multivariate analysis. The top variant for each locus is annotated by chromosome:base pair position (GRCh38). Lipid class names are listed in Figure 1. The *y*-axis is capped at 300. Two-sided *P*-values calculated using a linear-mixed-model (uv) and canonical correlation analysis (mv) are reported.

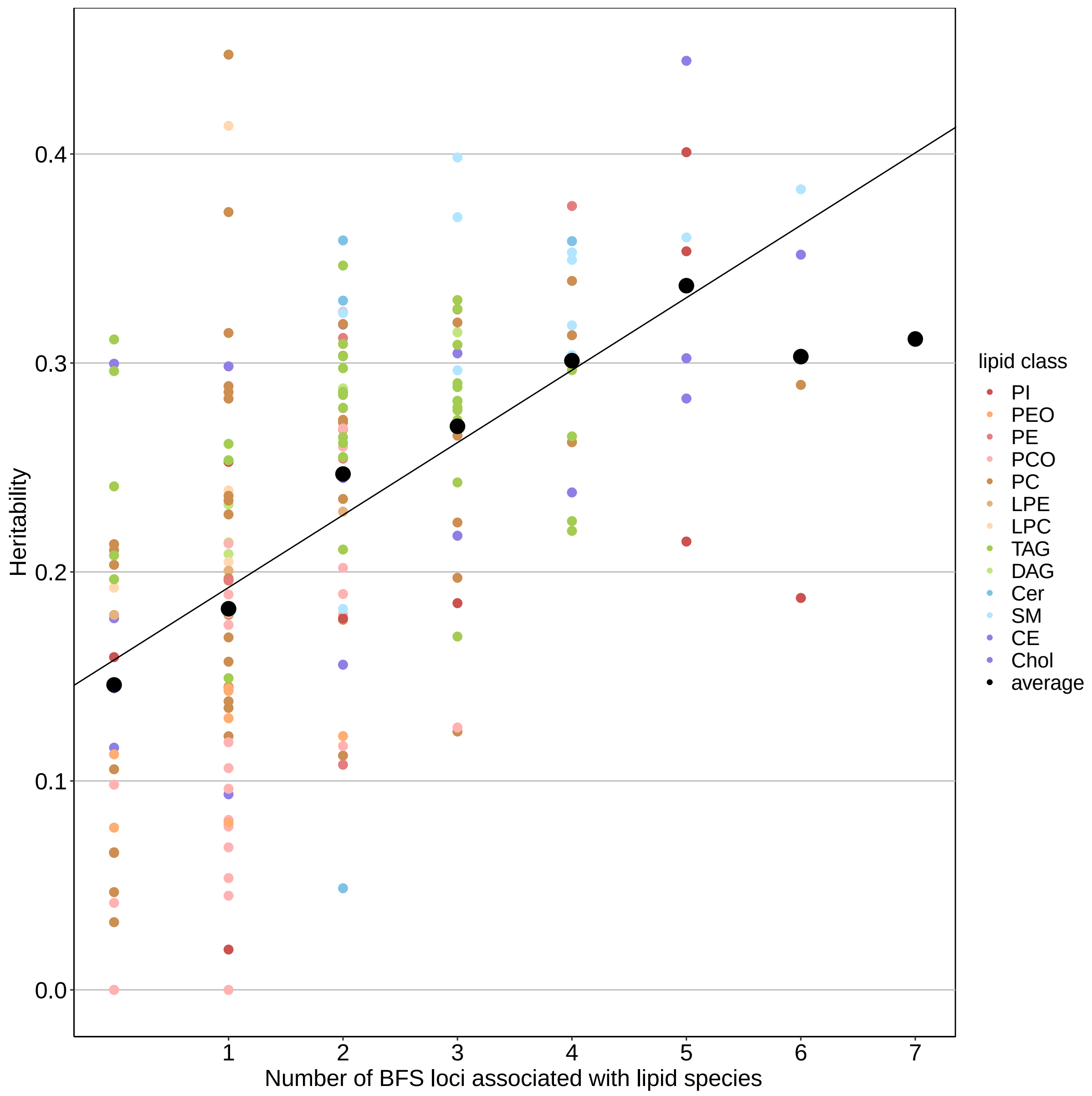

Supplementary Figure 10. Relationship between heritability estimates and the number of loci reaching the Bonferroni-corrected significance threshold of 7.352941e-10 per lipid species. The black line shows the linear regression fit (intercept=0.157927; se=0.009911, slope=0.034667; se=0.003888) with weights of the linear regression set to $1/se^2$. Larger black dots depict the average estimated heritability and smaller dots depict the individual heritability estimates colored by lipid class.

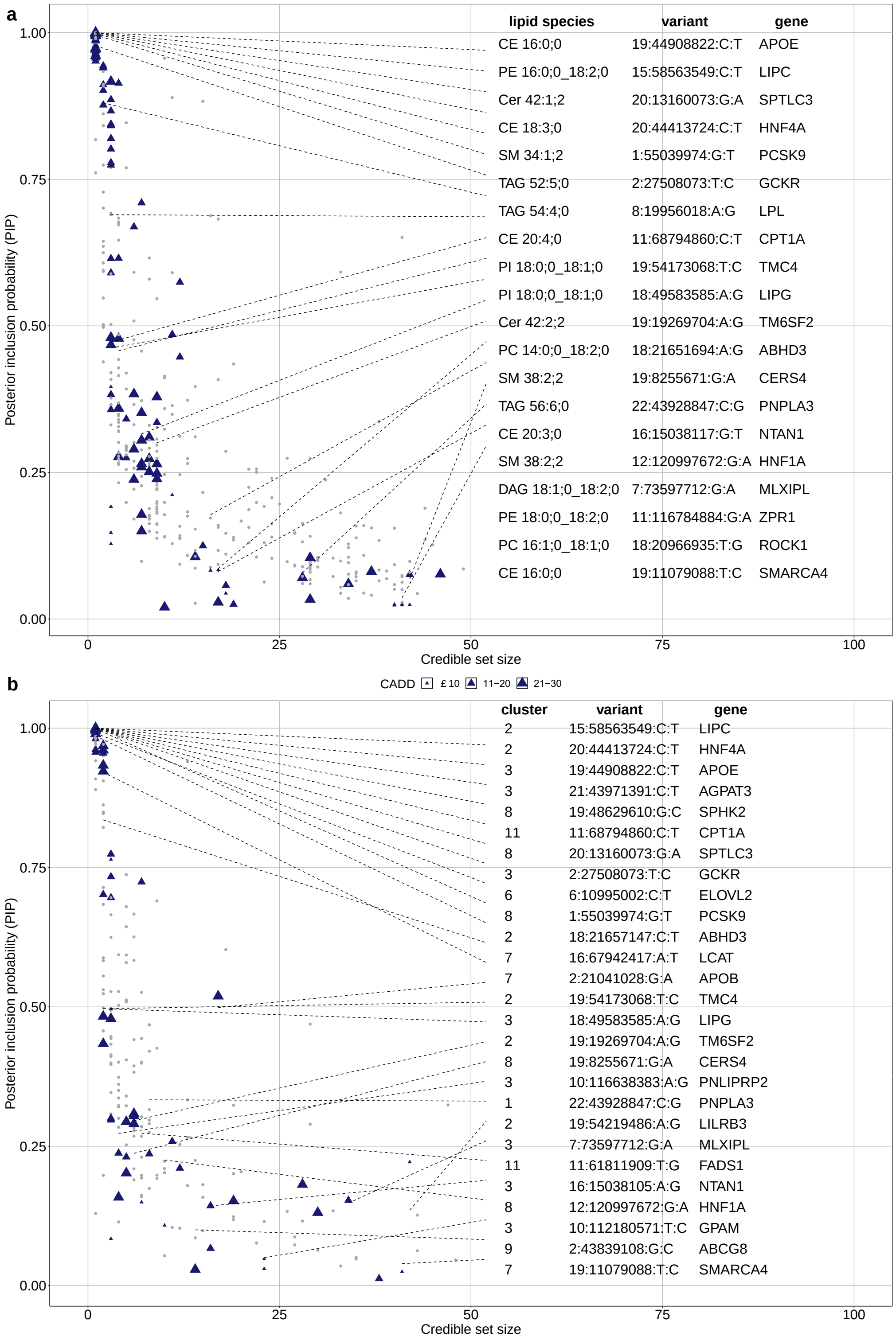

Supplementary Figure 11. Representative variants of informative 95% credible sets with credible set size < 50 and top PIP > 1% of BFS loci in (a) univariate GWAS (b) multivariate GWAS plotted by credible set size against PIP. Blue triangles are functional variants, other SNPs are shown as gray dots. The size of the triangles is proportional to the variants' CADD score. For loci for which functional variants were among the representative variants, the functional representative variant with the lowest GWAS *P*-value is annotated with the top associated trait, variant id, and gene from VEP.

a

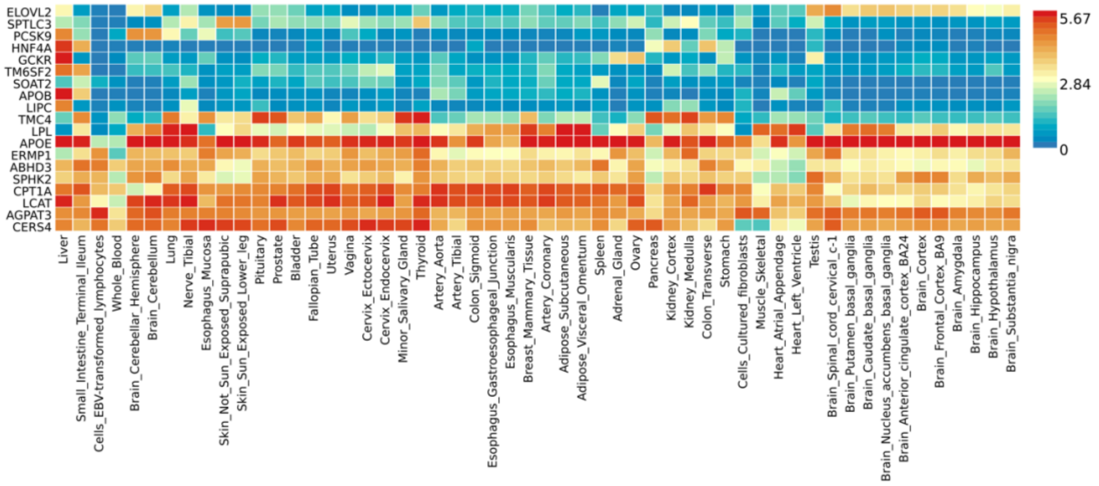

b

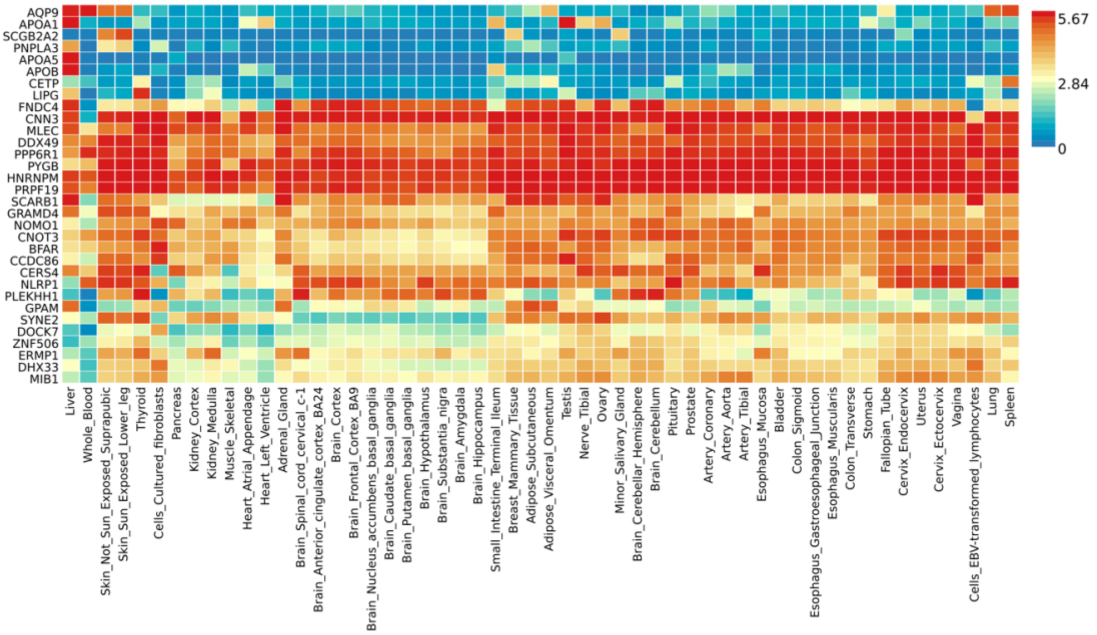

Supplementary Figure 12. Heatmap of expression level in GTEx tissues of genes prioritized by (a) functional variant approach and (b) FOCUS. The average log2 transformed expression value is shown.

a

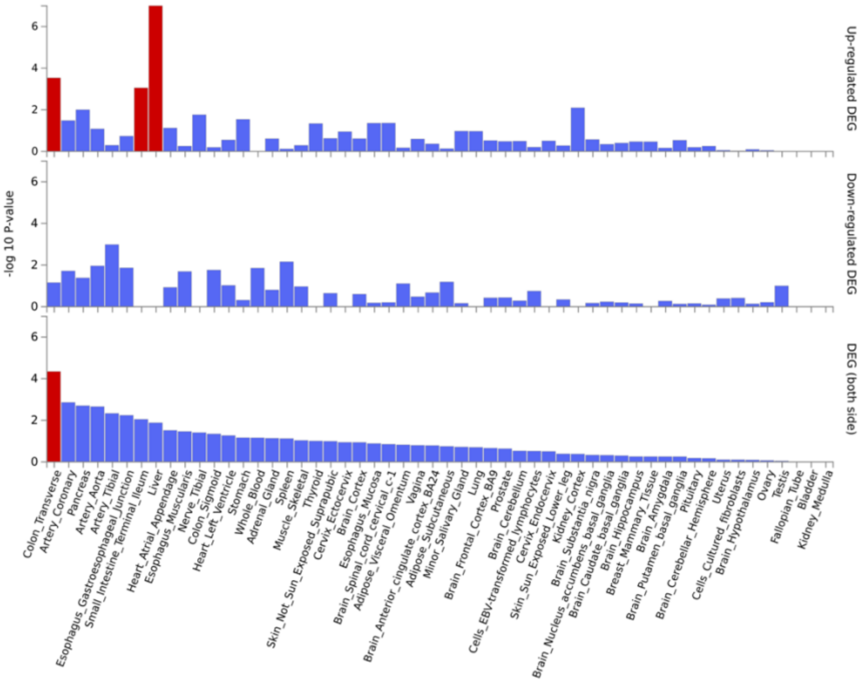

b

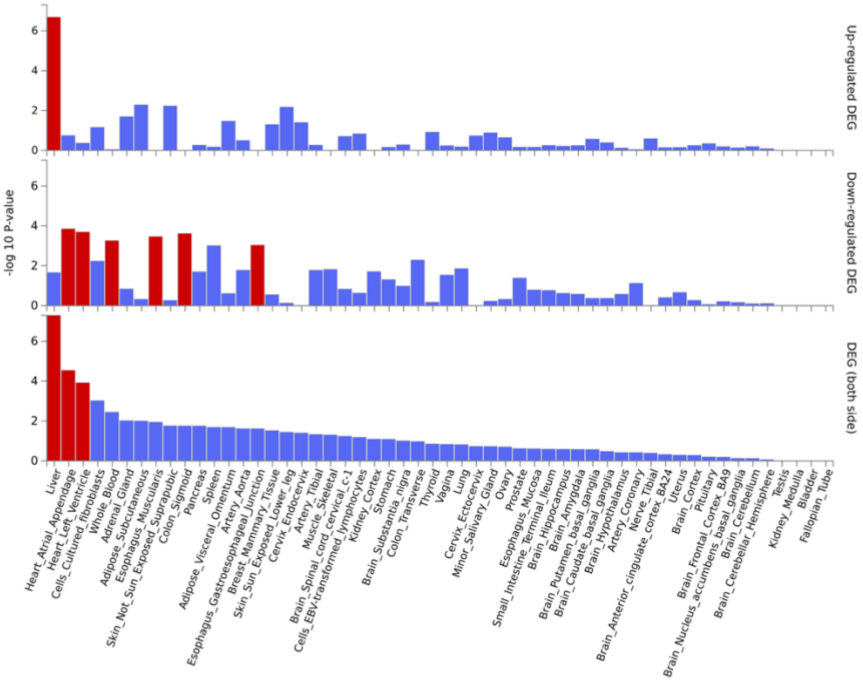

Supplementary Figure 13. Enrichment of differentially expressed genes (DEG) sets for genes prioritized by (a) functional variant approach and (b) FOCUS in 53 GTEx tissues. Significantly enriched DEG sets ($P \leq 0.05$ corrected for multiple testing) are highlighted in red. DEG sets were calculated by FUMA using two-sided *t*-tests for each gene per tissue against all other tissues. The DEG set in a given tissue is defined as genes with multiple testing-corrected *P*-value < 0.05 and absolute log fold change ≥ 0.58. The sign of the *t*-score is used to distinguish between upregulated and downregulated genes in a given tissue compared to other tissues. To test for enrichment of genes in DEG sets, genes are tested against each of the DEG sets using the hypergeometric test. Genes with average expression value > 1 in at least one of the tissues are utilized as background genes.

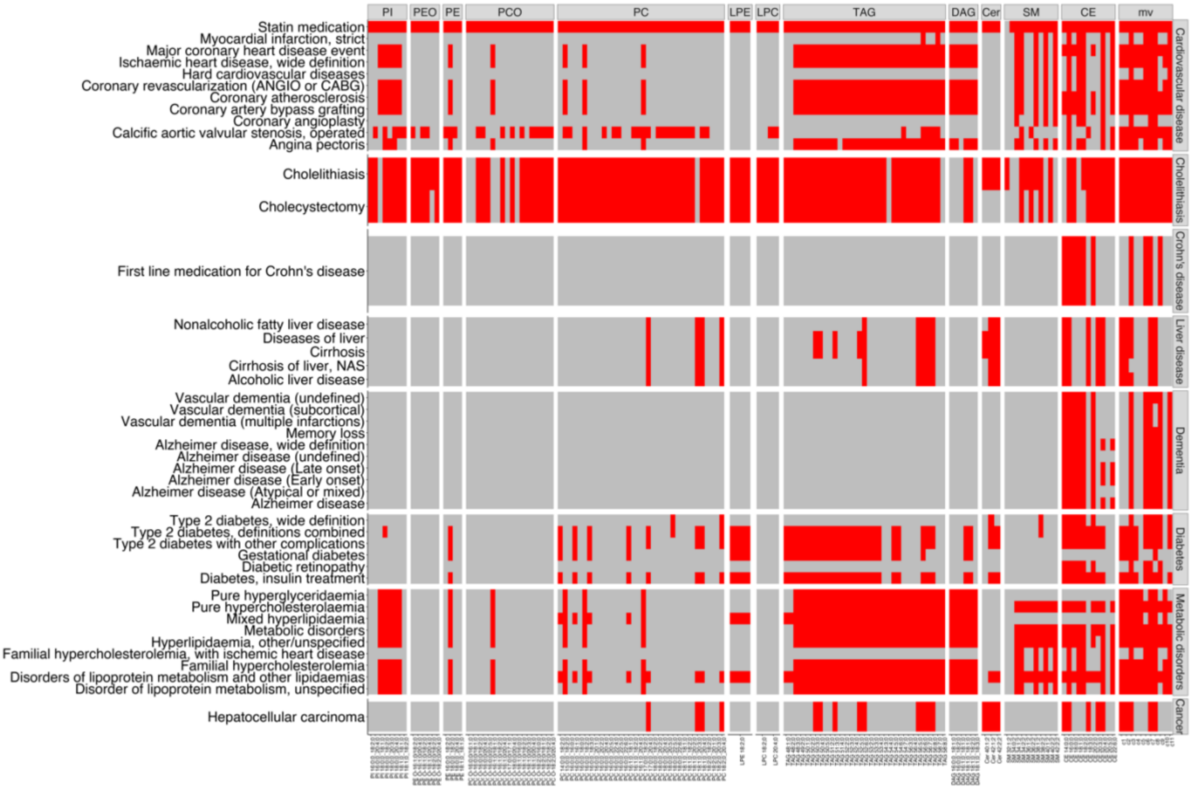

Supplementary Figure 14. Heatmap of PheWAS associations. Each entry in the heatmap represents a PheWAS endpoint of a disease endpoint (row), which is associated with variants among the lead variants or representative variants of a lipidome trait (column). Columns are split by lipid classes and rows are split by disease groups. Lead variants reaching the Bonferroni-corrected significance (BFS, uv: *P* < 7.352941e-10, mv: *P* < 4.545455e-9) and representative variants of BFS associations are considered. PheWAS associations reaching BFS (*P* < 5.24659e-11) are included. BFS associations are colored red. Two-sided *P*-values for lipidome traits were calculated using a linear-mixed-model (uv) and canonical correlation analysis (mv). Two-sided *P*-values for disease endpoints were calculated using mixed-model logistic regression.

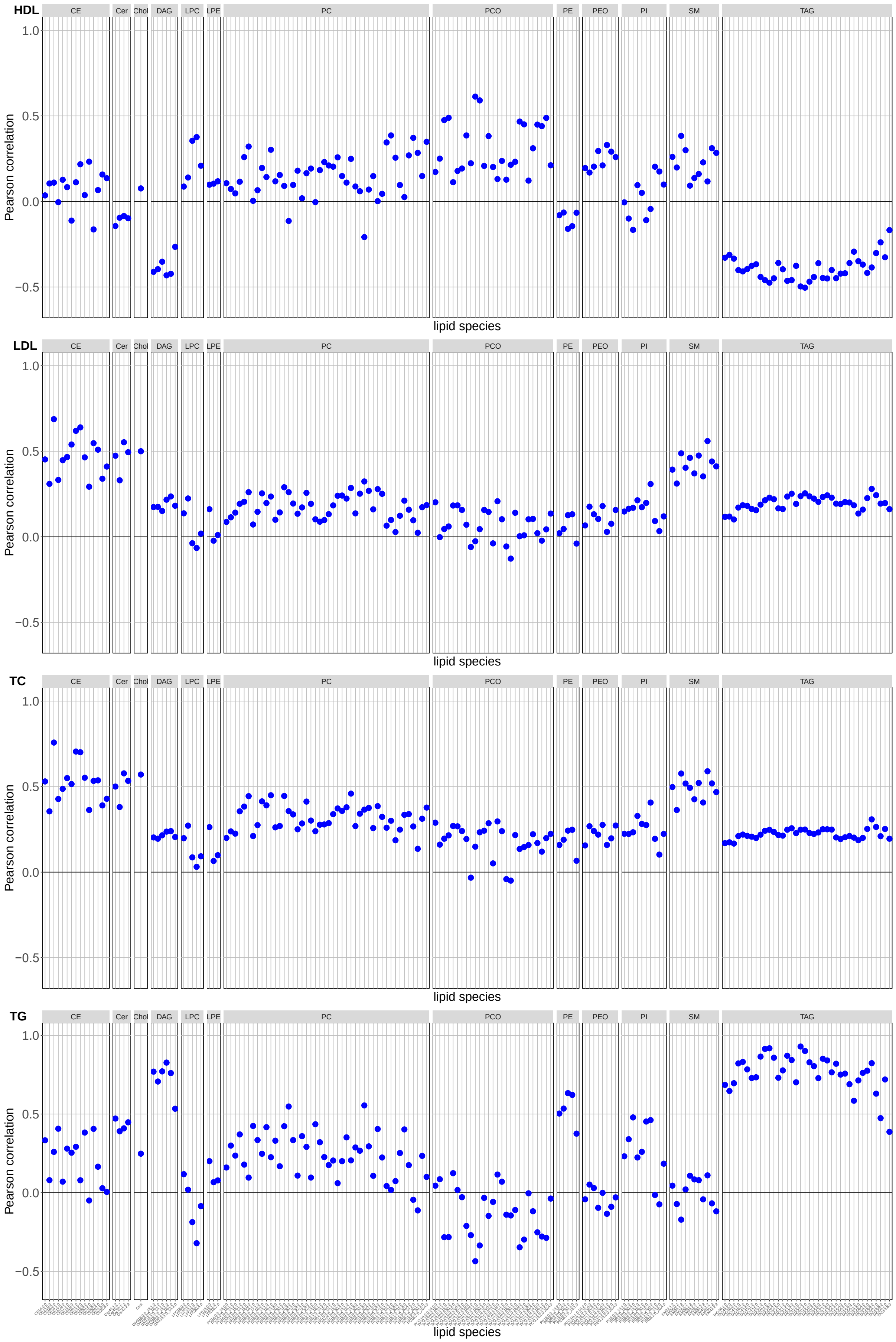

Supplementary Figure 15. Pairwise Pearson correlations of lipid species and standard lipids.

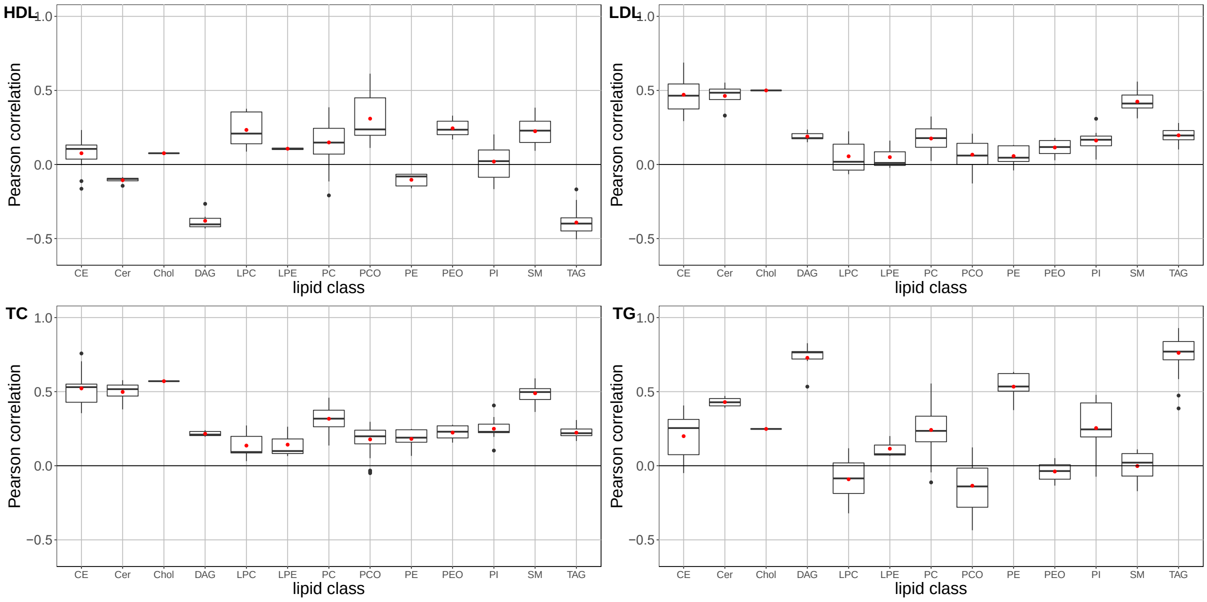

Supplementary Figure 16. Boxplots of pairwise Pearson correlations of lipid species of different lipid classes and standard lipids. The boxplots depict the mean Pearson correlation (middle line), interquartile range (IQR) defining the bounds of the box, and whiskers extending to the largest/smallest values no further than 1.5 times the IQR. Data beyond the end of whiskers are plotted individually. The number of lipid species per lipid class is: 15 (CE), 4 (Cer), 1 (Chol), 6 (DAG), 5 (LPC), 3 (LPE), 46 (PC), 27 (PCO), 5 (PE), 8 (PEO), 10 (PI), 11 (SM), 38 (TAG).

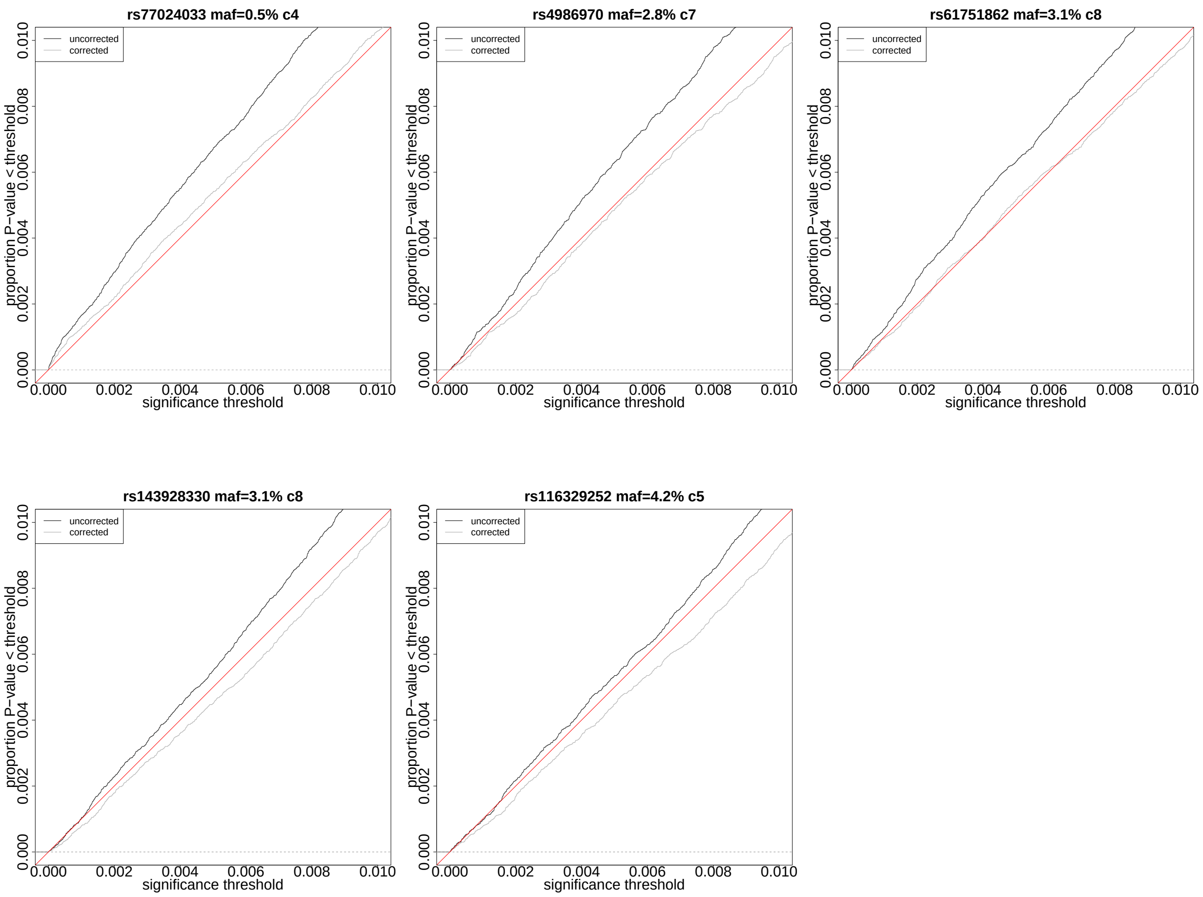

Supplementary Figure 17. Empirical cumulative distribution function (ECDF) plots of simulated *P*-values for rare or low-frequency variants reaching GWS in a multivariate analysis, which did not reach GWS in a univariate GWAS. The ECDF for uncorrected *P*-values is depicted by a black line and corrected *P*-values by a grey line. Red line shows the diagonal.
