# Supplementary material for: Genome-wide association analysis of plasma lipidome identifies 495 genetic associations
Source: Nat Commun. 2023 Oct 31;14:6934. doi: 10.1038/s41467-023-42532-8 (PMC10618167; doi:10.1038/s41467-023-42532-8)
Supplement: Supplementary file 3 — Description of Additional Supplementary Files [file 41467_2023_42532_MOESM3_ESM.pdf]

Description of Additional Supplementary Files

File Name: Supplementary Data 1
Description: Lipid species information and FinnGen consortium members.

File Name: Supplementary Data 2
Description: Loci reaching genome-wide significance (GWS) in univariate or multivariate GWAS.

File Name: Supplementary Data 3
Description: Results of fine-mapping analysis by FINEMAP.

File Name: Supplementary Data 4
Description: Results of gene prioritization and gene set enrichment analysis.

File Name: Supplementary Data 5
Description: Results of PheWAS.

File Name: Supplementary Data 6
Description: Results of colocalization analysis and lipid species associations with CAD loci.

File Name: Supplementary Data 7
Description: Associated variants from previous standard lipids and lipidome studies.
