# Supplementary material for: Genome-wide association analysis of plasma lipidome identifies 495 genetic associations
Source: Nat Commun. 2023 Oct 31;14:6934. doi: 10.1038/s41467-023-42532-8 (PMC10618167; doi:10.1038/s41467-023-42532-8)
Supplement: Supplementary file 11 — Reporting Summary [file 41467_2023_42532_MOESM11_ESM.pdf]

nature portfolio

Corresponding author(s): Matti Pirinen

Last updated by author(s): Oct 10, 2023

Reporting Summary

Nature Portfolio wishes to improve the reproducibility of the work that we publish. This form provides structure for consistency and transparency in reporting. For further information on Nature Portfolio policies, see our Editorial Policies and the Editorial Policy Checklist.

Statistics

For all statistical analyses, confirm that the following items are present in the figure legend, table legend, main text, or Methods section.

n/a	Confirmed	
☐	☒	The exact sample size (*n*) for each experimental group/condition, given as a discrete number and unit of measurement
☐	☒	A statement on whether measurements were taken from distinct samples or whether the same sample was measured repeatedly
☐	☒	The statistical test(s) used AND whether they are one- or two-sided<br>*Only common tests should be described solely by name; describe more complex techniques in the Methods section.*
☐	☒	A description of all covariates tested
☐	☒	A description of any assumptions or corrections, such as tests of normality and adjustment for multiple comparisons
☐	☒	A full description of the statistical parameters including central tendency (e.g. means) or other basic estimates (e.g. regression coefficient) AND variation (e.g. standard deviation) or associated estimates of uncertainty (e.g. confidence intervals)
☐	☒	For null hypothesis testing, the test statistic (e.g. *F*, *t*, *r*) with confidence intervals, effect sizes, degrees of freedom and *P* value noted<br>*Give P values as exact values whenever suitable.*
☐	☒	For Bayesian analysis, information on the choice of priors and Markov chain Monte Carlo settings
☒	☐	For hierarchical and complex designs, identification of the appropriate level for tests and full reporting of outcomes
☐	☒	Estimates of effect sizes (e.g. Cohen's *d*, Pearson's *r*), indicating how they were calculated

Our web collection on statistics for biologists contains articles on many of the points above.

Software and code

Policy information about availability of computer code

Data collection	Lipid identification software and data management system developed in-house by Lipotype GmbH (Herzog et al. 2011 doi: 10.1186/gb-2011-12-1-r8, Herzog et al. 2012 10.1371/journal.pone.0029851).
Data analysis	The following softwares were used for imputation and association analyses: Eagle v2.3.5, Beagle v4.1, biMM (release from 03.03.2017), MMM v1.01, R package metaCCA v1.13.1, fastGWA from GCTA v1.93.2. Softwares PLINK v1.9, MetaPhat (release from 01.07.2020), LDstore2, FINEMAP v1.4, FOCUS v0.7 and FUMA v1.3.7 were used for statistical analyses. LiftOver was used for lifting over chromosomal positions, CADD v1.6, Variant Annotation Integrator, LDlink release 5.3.3, SNP-nexus v4 and VEP v103.1 were used for variant annotation. R version 3.4.1 was used for data transformations, plotting of the results and statistical analyses including packages ggplot2, ggh4x, ggpubr, corrplot, ggcorrplot, car and data.table.

For manuscripts utilizing custom algorithms or software that are central to the research but not yet described in published literature, software must be made available to editors and reviewers. We strongly encourage code deposition in a community repository (e.g. GitHub). See the Nature Portfolio guidelines for submitting code & software for further information.

Data

Policy information about availability of data

All manuscripts must include a data availability statement. This statement should provide the following information, where applicable:

- Accession codes, unique identifiers, or web links for publicly available datasets
- A description of any restrictions on data availability
- For clinical datasets or third party data, please ensure that the statement adheres to our policy

The univariate GWAS summary statistics generated in this study have been deposited in the GWAS catalog under accession codes GCST90277238-GCST90277416 [http://ftp.ebi.ac.uk/pub/databases/gwas/summary_statistics/GCST90277001-GCST90278000/GCST90277238/].
The DNA, blood, serum, and plasma samples of the GeneRISK study participants, in addition to their demographic information, health, genotype, and lipidomics data are stored in the THL Biobank [https://thl.fi/en/web/thl-biobank/for-researchers/sample-collections/generisk-study]. The GeneRISK data are available under restricted access via procedures outlined in the Finnish Biobank Act and access can be obtained for biomedical research by contacting admin.biobank@thl.fi. A response to requests will be received within three weeks. Researchers may use the data only for purposes described in the application and are allowed to share the data with others only with a written approval from the THL Biobank.

Human research participants

Policy information about studies involving human research participants and Sex and Gender in Research.

Reporting on sex and gender	The cohort includes 7,292 participants (4,642 female sex, 2,624 male sex). Sex was determined based on self-reporting. Sex was included as a covariate in GWAS.
Population characteristics	The mean age (standard deviation) of female and male participants was 55.7 (5.7) and 55.9 (5.9). Of the participants with female and male sex, 432 (9.3%) and 373 (14.2%), respectively, used lipid-lowering medication.
Recruitment	The participants were recruited from Southern Finland during 2015-2017 at age of 45-66 years. Partipants were recruited from the Kymenlaakso province in South-Eastern Finland by identifying 4,857 individuals from the population register at random and inviting them by mail. Further 1,369 individuals were recruited from customers of Helsinki and Turku offices of a private and occupational health care provider. Additionally, online advertising was used to recruit 1,116 blood donors. Individuals under guardianship, with previous history of Atherosclerotic Cardiovascular Disease and pregnant women were excluded from the study.
Ethics oversight	The GeneRISK study was carried out according to the principles of the Helsinki declaration and the Council of Europe's (COE) Convention of Human Rights and Biomedicine. All study participants gave their informed consent to participate in the study. The study protocols were approved by The Hospital District of Helsinki and Uusimaa Coordinating Ethics committees (approval No. 281/13/03/00/14 (GeneRISK)).<br>Patients and control subjects in FinnGen provided informed consent for biobank research, based on the Finnish Biobank Act. Alternatively, separate research cohorts, collected prior the Finnish Biobank Act came into effect (in September 2013) and start of FinnGen (August 2017), were collected based on study-specific consents and later transferred to the Finnish biobanks after approval by Fimea (Finnish Medicines Agency), the National Supervisory Authority for Welfare and Health. Recruitment protocols followed the biobank protocols approved by Fimea. The Coordinating Ethics Committee of the Hospital District of Helsinki and Uusimaa (HUS) statement number for the FinnGen study is Nr HUS/990/2017.<br><br>The FinnGen study is approved by Finnish Institute for Health and Welfare (permit numbers: THL/2031/6.02.00/2017, THL/1101/5.05.00/2017, THL/341/6.02.00/2018, THL/2222/6.02.00/2018, THL/283/6.02.00/2019, THL/1721/5.05.00/2019 and THL/1524/5.05.00/2020), Digital and population data service agency (permit numbers: VRK43431/2017-3, VRK/6909/2018-3, VRK/4415/2019-3), the Social Insurance Institution (permit numbers: KELA 58/522/2017, KELA 131/522/2018, KELA 70/522/2019, KELA 98/522/2019, KELA 134/522/2019, KELA 138/522/2019, KELA 2/522/2020, KELA 16/522/2020), Findata permit numbers THL/2364/14.02/2020, THL/4055/14.06.00/2020,,THL/3433/14.06.00/2020, THL/4432/14.06/2020, THL/5189/14.06/2020, THL/5894/14.06.00/2020, THL/6619/14.06.00/2020, THL/209/14.06.00/2021, THL/688/14.06.00/2021, THL/1284/14.06.00/2021, THL/1965/14.06.00/2021, THL/5546/14.02.00/2020, THL/2658/14.06.00/2021, THL/4235/14.06.00/202, Statistics Finland (permit numbers: TK-53-1041-17 and TK/143/07.03.00/2020 (earlier TK-53-90-20) TK/1735/07.03.00/2021, TK/3112/07.03.00/2021) and Finnish Registry for Kidney Diseases permission/extract from the meeting minutes on 4th July 2019.<br><br>The Biobank Access Decisions for FinnGen samples and data utilized in FinnGen Data Freeze 9 include: THL Biobank BB2017_55, BB2017_111, BB2018_19, BB_2018_34, BB_2018_67, BB2018_71, BB2019_7, BB2019_8, BB2019_26, BB2020_1, Finnish Red Cross Blood Service Biobank 7.12.2017, Helsinki Biobank HUS/359/2017, HUS/248/2020, Auria Biobank AB17-5154 and amendment #1 (August 17 2020), AB20-5926 and amendment #1 (April 23 2020) and it´s modification (Sep 22 2021), Biobank Borealis of Northern Finland_2017_1013, Biobank of Eastern Finland 1186/2018 and amendment 22 § /2020, Finnish Clinical Biobank Tampere MH0004 and amendments (21.02.2020 & 06.10.2020), Central Finland Biobank 1-2017, and Terveystalo Biobank STB 2018001 and amendment 25th Aug 2020.

Note that full information on the approval of the study protocol must also be provided in the manuscript.

Field-specific reporting

Please select the one below that is the best fit for your research. If you are not sure, read the appropriate sections before making your selection.

☒ Life sciences ☐ Behavioural & social sciences ☐ Ecological, evolutionary & environmental sciences

For a reference copy of the document with all sections, see nature.com/documents/nr-reporting-summary-flat.pdf

Life sciences study design

All studies must disclose on these points even when the disclosure is negative.

Sample size	We used data from the GeneRISK cohort provided by THL biobank. We used all such individuals from the GeneRISK cohort who had both lipidome measurements and genotype data available. This resulted in the sample size of 7,174 individuals. As we did not collect any samples in this study, the final sample size was determined by the availability of the samples in THL biobank. The sample size is sufficient to find many genome-wide significant associations (495).
Data exclusions	Lipids with a high signal-to-noise ratio (> 5) and amounts at least 5-fold higher than corresponding blank samples were included. Lipid species detected in more than 70% of the samples were included. Samples with very low total lipid content and with > 30% of 179 lipids missing were excluded. Individuals with non-Finnish ancestry or birthplace were removed. Samples (N=30) with extreme heterozygosity (beyond ± 4 s.d) were excluded. Very rare variants (MAF < 0.002) and variants with low imputation quality (INFO < 0.8) were excluded.
Replication	We systematically assessed whether each of our finding was a replication of any previously reported lipid locus. We did not have available additional data to attempt a further replication of our findings.
Randomization	Participants were not allocated to different experimental groups in this study.
Blinding	Blinding is not relevant to this study as this is an observational study.

Reporting for specific materials, systems and methods

We require information from authors about some types of materials, experimental systems and methods used in many studies. Here, indicate whether each material, system or method listed is relevant to your study. If you are not sure if a list item applies to your research, read the appropriate section before selecting a response.

Materials & experimental systems

n/a	Involved in the study	
☒	☐	Antibodies
☒	☐	Eukaryotic cell lines
☒	☐	Palaeontology and archaeology
☒	☐	Animals and other organisms
☒	☐	Clinical data
☒	☐	Dual use research of concern

Methods

n/a	Involved in the study	
☒	☐	ChIP-seq
☒	☐	Flow cytometry
☒	☐	MRI-based neuroimaging
